# A Boolean-based systems biology approach to predict novel genes associated with cancer: Application to colorectal cancer

**DOI:** 10.1186/1752-0509-5-35

**Published:** 2011-02-26

**Authors:** Shivashankar H Nagaraj, Antonio Reverter

**Affiliations:** 1 Computational and Systems Biology, Commonwealth Scientific and Industrial Research Organisation (CSIRO), Division of Livestock Industries, Queensland Bioscience Precinct, 306 Carmody Road, St. Lucia, Brisbane, Queensland 4067, Australia

## Abstract

**Background:**

Cancer has remarkable complexity at the molecular level, with multiple genes, proteins, pathways and regulatory interconnections being affected. We introduce a systems biology approach to study cancer that formally integrates the available genetic, transcriptomic, epigenetic and molecular knowledge on cancer biology and, as a proof of concept, we apply it to colorectal cancer.

**Results:**

We first classified all the genes in the human genome into cancer-associated and non-cancer-associated genes based on extensive literature mining. We then selected a set of functional attributes proven to be highly relevant to cancer biology that includes protein kinases, secreted proteins, transcription factors, post-translational modifications of proteins, DNA methylation and tissue specificity. These cancer-associated genes were used to extract 'common cancer fingerprints' through these molecular attributes, and a Boolean logic was implemented in such a way that both the expression data and functional attributes could be rationally integrated, allowing for the generation of a guilt-by-association algorithm to identify novel cancer-associated genes. Finally, these candidate genes are interlaced with the known cancer-related genes in a network analysis aimed at identifying highly conserved gene interactions that impact cancer outcome. We demonstrate the effectiveness of this approach using colorectal cancer as a test case and identify several novel candidate genes that are classified according to their functional attributes. These genes include the following: 1) secreted proteins as potential biomarkers for the early detection of colorectal cancer (*FXYD1*, *GUCA2B, REG3A*); 2) kinases as potential drug candidates to prevent tumor growth (*CDC42BPB, EPHB3, TRPM6*); and 3) potential oncogenic transcription factors (*CDK8*, *MEF2C, ZIC2*).

**Conclusion:**

We argue that this is a holistic approach that faithfully mimics cancer characteristics, efficiently predicts novel cancer-associated genes and has universal applicability to the study and advancement of cancer research.

## Background

Cancer is a complex genetic disease that exhibits remarkable complexity at the molecular level with multiple genes, proteins and pathways and regulatory interconnections being affected. Treating cancer is equally complex and depends on a number of factors, including environmental factors, early detection, chemotherapy and surgery. Cancer is being recognized as a systems biology disease [1,2], as illustrated by multiple studies that include molecular data integration and network and pathway analyses in a genome-wide fashion. Such studies have advanced cancer research by providing a global view of cancer biology as molecular circuitry rather than the dysregulation of a single gene or pathway. For instance, reverse-engineering of gene networks derived from expression profiles was used to study prostate cancer [3], from which the androgen-receptor (AR) emerged as the top candidate marker to detect the aggressiveness of prostate cancers. Similarly, sub-networks were proposed as potential markers rather than individual genes to distinguish metastatic from non-metastatic tumors in a breast cancer study [4]. The authors in this study argue that sub-network markers are more reproducible than individual marker genes selected without network information and that they achieve higher accuracy in the classification of metastatic versus non-metastatic tumor signaling. Using genome-wide dysregulated interaction data in B-cell lymphomas, novel oncogenes have been predicted *in-silico *[5]. Finally, taking a signaling-pathway approach, a map of a human cancer signaling network was built [6] by integrating cancer signaling pathways with cancer-associated, genetically and epigenetically altered genes.

Gene expression profiling has been widely used to investigate the molecular circuitry of cancer. In particular, DNA microarrays have been used in almost all of the main cancers and promise to change the way cancer is diagnosed, classified and treated [1]. However, expression analyses often result in hundreds of outliers, or differentially expressed genes between normal and cancer cells or across time points [2]. Owing to the large number of candidate genes, several different hypotheses can be generated to explain the variation in the expression patterns for a given study. In addition, the preferential expressions of some tissue-specific genes present additional challenges in expression data analyses. Nevertheless, recent systems approaches have attempted to prioritize differentially expressed genes by overlaying expression data with molecular data, such as interaction data [3], metabolic data [4] and phenotypic data [5].

Human malignancies are not just confined to genes and gene products, but also include epigenetic modifications such as DNA methylation and chromosomal aberrations. However, in order to effectively capture the properties that emerge in a complex disease, we need analytical methods that provide a robust framework to formally integrate prior knowledge of the biological attributes with the experimental data. The simplest heuristic will search for novel genes with a profile, in terms of differential expression and/or network connectivity, similar to those for which an association to disease has been well established (see, for instance, the approaches of [7,8]).

Boolean logic has been found to be optimal for such tasks. Within the context of cancer, Mukherjee and Speed [9] show how a series of biological attributes including ligands, receptors and cytosolic proteins, can be included in the network inference. More recently, Mukherjee and co-workers [10] introduced an approach based on sparse Boolean functions and applied it to the responsiveness of breast cancer cell lines to an anti-cancer agent. In addition, large scale literature-based Boolean models have been used to study apoptosis pathways as well as pathways connected with them.

In this study, we propose a systems biology approach to predict disease-associated genes that are either not previously reported (novel) or poorly characterized and using colorectal cancer as a case study. To achieve this goal, we first implemented a Boolean logic schema derived from cancer-associated genes and developed a guilt-by-association (GBA) algorithm, which is subsequently applied in a genome-wide fashion. Although gene expression data are central to this approach, other biologically relevant functional attributes, such as tissue specificity, are treated as equally important in the Boolean logic informing the GBA algorithm. Finally, novel cancer-associated genes are interlaced with the known cancer-related genes in a weighted network circuitry aimed at identifying highly conserved gene interactions that impact cancer outcome.

## Results and Discussion

### Overview of the systems biology approach

**Figure 1:**
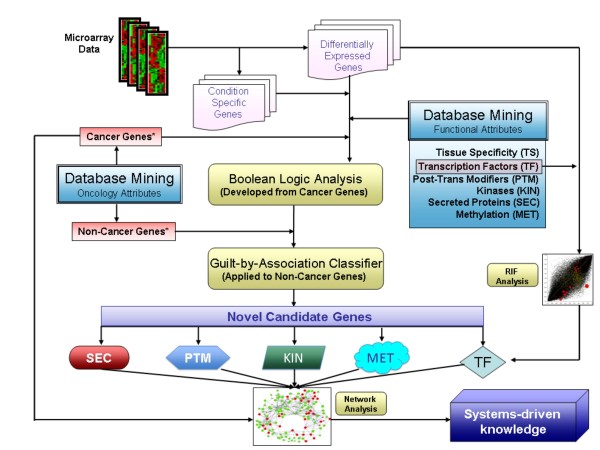
**The schema for the identification of novel genes associated with complex diseases**. The expression profiles from the cancer data are analyzed to predict differentially expressed and condition-specific genes. The functional attributes over-represented in cancer are selected and representative datasets from public resources mined. The common cancer fingerprints from cancer-associated genes are processed through Boolean logic to develop a guilt-by-association classifier which, applied to non-cancer-associated genes, predicts novel candidate cancer-associated genes. Finally, novel candidate genes are further analyzed using network theory approaches.

Figure 1 shows the schema of the proposed analytical approach. The first phase deals with the analysis of gene expression data to obtain a list of differentially expressed and condition specific genes. Conceptually, differentially expression differs from condition specificity in that the former requires the postulation of a contrast of interest while the latter enriches for genes that are preferentially expressed in one of the (potentially many) experimental conditions being considered. Nevertheless, the expectation is for a substantial overlap in the genes identified between either criterion. In the second phase, public databases are mined to compile a list of cancer-associated genes, non cancer-associated genes and functional attributes that are of relevance in the context of cancer. We considered a total of six functional attributes as follows: tissue specificity (TS), transcription factors (TF), post-translational modifications (PTM), kinases (KIN), secreted proteins (SEC) and CpG island methylation (MET)(see Additional File 1 for rationale behind choosing these attributes). Table 1 summarizes the general characteristics of the functional attributes with a few prototypic examples of representative genes. Additional File 2 provides the list of 749 cancer-associated genes that we compiled within each attribute. These features were selected based also on the fact that there is a strong functional interconnection among them and therefore we see the overlapping of these genes across attributes.

**Table 1:** Overview of the genetic, epigenetic and molecular information used in this study

Functional Attribute	Role in Cancer	Potential application	Examples	Data source	Reference
Cancer associated genes	Genes with at least 2 mutations in causally implicated in cancer. Includes oncogenes, tumor suppressor genes	Potential drug targets and diagnostic or prognostic markers	Oncogenes: *BCL2, c-Jun, ERG, ERBB2, RAS, c-MYC, c-SRC* Tumor Suppressor Genes: *RB1, P53, APC, BRCA-1*, *BRCA-2*	http://www.sanger.ac.uk/genetics/CGP/Census/ http://hprd.org/ Reviews: (Futreal et al, 2004; Hahn et al, 2002; Mitelman, 2000; Vogelstein et al, 2004)	NA

Non-cancer associated genes	There is no previous report of any causal mutation.	If cancer association is established, these genes are either potential drug targets and diagnostic or prognostic markers	*AMN, B3GNTL1, CDC42BPB* *S100A9, TRPM6, VNN1, ZIC2*	NCBI - Human Genome http://www.ncbi.nlm.nih.gov/projects/genome/guide/human/	NA

Kinases	More than 30% of cancer related genes are kinases and the most common domain that is encoded by cancer genes is the protein kinase domain	Drug targets through inhibitors	*c-Src, c-Abl, RAS*, mitogen activated protein (MAP) kinase, phosphotidylinositol-3-kinase (PI3K), *AKT*, and the epidermal growth factor receptor (EGFR)	Human Kinome Consortium http://kinase.com/human/kinome/	[15] [17,51]

Excretory - Secretory proteins	Malignant tumors secrete increased levels of ES proteins	non-invasive diagnostic or prognostic markers for early detection	alpha-fetoprotein, *CD44*, kallikrein 6, kallikrein 10, *MIC-1*	Secreted Protein Database (SPD) http://spd.cbi.pku.edu.cn/	[52,53] [54] [55]

Transcription factors	Overactivity of TFs at different stages of cancer is well documented and novel treatment strategies have been suggested for targeted inhibition of oncogenic TFs	Alternative therapeutic strategy, potential drug targets	*C-MYB, NF-kappaB, AP-1, STAT *and *ETS *transcription factors	Genomatix http://www.genomatix.de/	[15,56] [57] [58]

DNA Methylation	Methylation patterns are altered in cancer cells as shown in hypomethylation of oncogenes and hypermethylation of tumor suppressor resulting in gene silencing or gene inactivation	CpG island methylation could be used as a biomarker of malignant cells	*hMLH1, BRCA1, MGMT, p16(INK4a), p14(ARF), p15(INK4b, DAPK, APAF-1*	Human Colon Methylome from [29]	[27,59] [28] [60,61]

Post-translational modifications	Key proteins driving oncogenesis, Can undergo PTM Although Phosphoryltion is partially covered in kinases section, other PTMs such as glycosylation and ubiquitination reported to play a role in malignancies, are included separate functional gene attributes.		*BRCA1, EGFR, c-Src, c-Abl, RAS, TP53*	HPRD http://hprd.org/	[18] Burger and Seth, 2004)

The resulting set of variables (differentially expression, condition specificity, and the six functional attributed) are each binarized and used in a Boolean logic framework. The Boolean logic is then applied to cancer-associated genes to develop a GBA algorithm. When applied to non cancer-associated genes, the GBA algorithm preferentially ranks those genes whose behavior across all variables most mimics that of cancer-associated genes. Finally, in order to gain a global understanding of the novel candidate genes, we generate a series of gene co-expression networks. The resulting networks are surveyed with a focus on the interacting partners of candidate genes and within the context of the original functional attributes.

### Differentially expressed and condition specific genes

**Figure 2:**
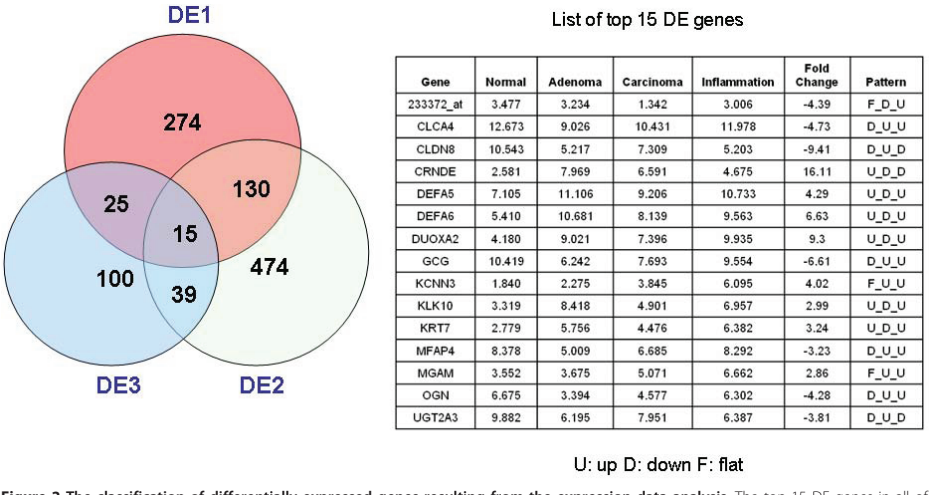
**The classification of differentially expressed genes resulting from the expression data analysis**. The top 15 DE genes in all of the three categories are tabulated with their expression values in normal, adenoma, carcinoma and inflammation.

We explored three measures of differential expression (DE1 = Carcinoma - Normal; DE2 = Carcinoma - Adenoma; and DE3 = Carcinoma - Inflammation) and identified 444, 658 and 179 differentially expressed genes for DE1, DE2, and DE3, respectively. We observed several overlaps among the three differentially expressed gene categories, and 15 genes were found to be differentially expressed in all three categories (Figure 2). Among them, we highlight *CLCA4, CRNDE, DEFA5, DUOXA2, GCG, KLK10*, and *UGT2A3*. In particular, *CRNDE *(colorectal neoplasia differentially expressed) was the most differentially expressed (up-regulated) gene with a 16-fold change in expression. *CRNDE *gene is localized to chromosome 16 (16q12.2) and is poorly characterized with no functional information on its role in colorectal cancer except its differential expression from the EST data (UniGene Id: 167645). Another differentially expressed gene *KLK10 *is a member of the kallikrein gene family which is well documented biomarker for the detection of colon, ovarian and pancreatic cancers [8,11].

In addition, we identified 83, 61, 23, and 48 condition specific genes for Normal, Adenoma, Carcinoma and Inflammation, respectively. Among these genes, 23 were found to be specific to carcinoma (CS3) (see Additional File 1 Table S1). Notably, *CCDC3, EREG, IL6, PAPPA*, *SERPINE1, TFPI2 *and *THBS2 *are a few examples of the condition specific genes that appeared as top candidates. In particular, *CCDC3 *(coiled-coil domain containing 3) and *TFPI2 *(tissue factor pathway inhibitor 2) genes were the most carcinoma-specific genes.*TFPI2 *has been proposed to be a tumor suppressor gene as it's frequently methylated in colorectal cancer [7]. The *CCDC3 *encoded protein is predicted to be localized to extracellular matrix [12] with no previous association with colorectal cancer. Higher IL-6 levels might be prognostic indicator in colorectal cancer as they are associated with increasing tumor stages and tumor size, with metastasis and decreased survival [13].

Expression-profiling analyses often result in hundreds of candidate genes. The challenge is exacerbated when the expression data are gathered at different time points or in multiple conditions, as in the current study with a number of differentially expressed and condition specific genes. Nevertheless, it is a common practice to stop the *in-silico *expression analysis with the list of outliers and select one or more genes for experimental characterization based on the underlying biology. Often, expression data analyses are accompanied by downstream bioinformatics investigations such as Gene Ontology (GO) enrichment, pathway mapping and network reconstruction. It is also believed that expression data are not sufficient to accurately reconstruct biological networks [14] and that the incorporation of additional biological data is required to constrain the number of plausible hypotheses. We approached this challenge by first identifying the most relevant functional attributes that has been well documented in cancer and then extracting this information to build a Boolean logic.

### Boolean logic to develop a guilt-by-association (GBA) algorithm

We developed a model to infer a gene's association to cancer. The model accommodates biologically motivated semantics into a Boolean logic schema, but is of a probabilistic nature, allowing it to efficiently and effectively accommodate noise in biological concepts and data when ranking candidate genes (see Methods).

We trained the model from data based on the behavior of the cancer-associated genes across 13 binarized Boolean variables: the three measures of differential expression (whether or not a gene was differentially expressed in each of the three contrasts), the four measures of condition specificity (similarly binarized), and the six cancer-biology attributes as previously described. At least one of the 13 variables was assigned to 530 of the 749 cancer-associated genes. These were used to construct a probabilistic Boolean truth table (Additional File 3) with 70 combinations (out of a total of 2^13 ^= 8192 possible combinations).

The trained model is efficient in weighing each attribute based on firmly established principles in cancer biology. For instance, more than 30% of the cancer-associated genes encode protein kinases [15] and this information is implemented 'as is'. In addition the proportion of kinases that undergo a PTM is also stored in the model and applied to non cancer-associated genes to capture similar kinases that harbor PTM but are strongly controlled by differential expression or condition specific properties in a given expression study. Furthermore, the flexibility of this method lies in its ability to simultaneously address different aspects of cancer. For example, the model predicts novel biomarkers by analyzing the genome-wide expression profiles and exclusively selecting secreted proteins as functional attributes. This will identify differentially expressed or condition specific secreted proteins expressed in blood/serum/urine.

**Figure 3:**
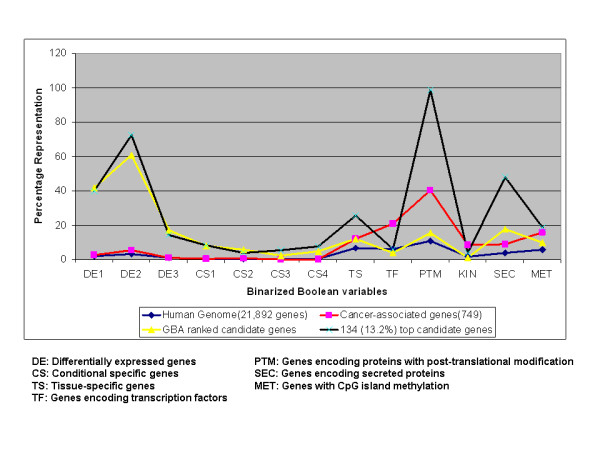
**Trends showing the distribution of genes across 13 binarized Boolean variables**. Four classes of genes were used for the comparison; i. all the genes in the human genome (21 892), ii. cancer-associated genes (749), iii. GBA ranked candidate genes candidate genes (1017) and iv. top candidate genes (134, 13.2%of the GBA ranked candidate genes). PTM and SEC classes are enriched in cancer-associated genes as well as in candidate genes category.

Next, we sought to obtain an overview of the representation of the 13 binarized Boolean variables across different gene classes which might provide additional insights into features of cancer genes in comparison to other genes. We selected the following four categories of genes: i. All the genes included in the analyses (n = 21 892); ii. The cancer-associated genes (n = 749), iii. The candidate genes processed by the GBA algorithm (n = 1017); and iv. The top candidate genes (n = 134, 13.2% of the genes processed by the GBA). Figure 3 shows the distribution of the four gene categories across the 13 variables. We observed enrichment for PTM and secreted proteins in both cancer-associated and top candidate genes. For instance, 40% of cancer-associated genes encoding protein had a PTM and 98% among the top candidate genes. Similarly, 8% and 47% of genes encoded for secreted proteins in cancer-associated genes and top candidate genes respectively. These results lead us to inspect the coverage for PTM and secreted protein both in cancer-associated genes as well as other genes as they contributed significantly in ranking the candidate genes. Additional File 1 Table S2 Shows exclusive and combined distribution of secreted proteins and PTM. Using chi-square test of independence, we examined the association of these two functional attributes and obtained a significant p-value of 3.713 E-06. This indicates that the association of PTM and secreted proteins either in combination or individually in cancer associated genes are significantly different compared to other genes. Finally, we note that the Boolean logic that gives rise to the GBA algorithm operates by exploiting the combinatorial nature of the 13 variables. Although, PTM are over-represented in both cancer-associated genes and hence candidate genes, their inclusion as one among five attributes was necessary as aberrant activation of signaling pathways drives cancer progression. For example, phosphorylation [16,17], glycosylation [18] and ubiquitination [19] have been documented to play key role in cancer progression.

### Computational validation of the analytical approach

We designed a two-step approach to ascertain the inferential validity of the proposed GBA. In the first step, we processed all genes through the Boolean logic using the previously developed probabilistic truth table. We found that known cancer genes received an average Boolean score of 0.219 (range: 0.002 to 0.687), compared to an average score of 0.081 (range: 0.000 to 0.589) for the other genes. This indicates that our Boolean logic yields a score to cancer genes that is on average 2.71-fold higher than that of candidate genes. This odds ratio was used as the threshold to be applied for the calibration in the second step of the validation.

**Figure 4:**
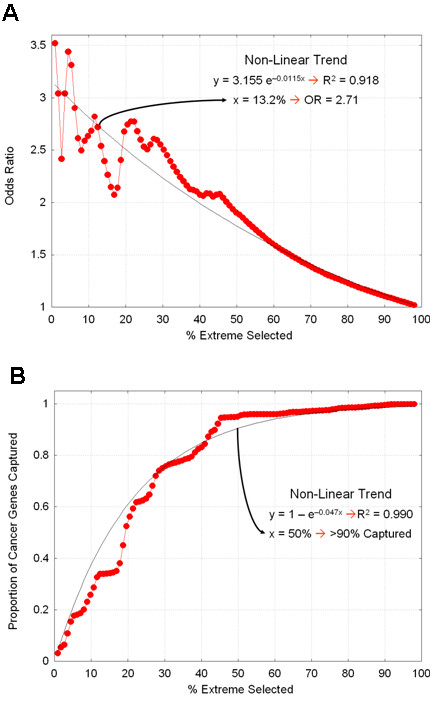
**Two-step computational validation approach to ascertain the inferential validity of the proposed GBA**. **4A **shows the ratio of the average Boolean score given to cancer genes over the average score given to the other genes. Candidate genes comprising the top 13.2% of genes guarantee a 2.71-fold over-representation of cancer genes. **4B**. Standard cross-validation in which the proportion of cancer-associated genes are compared to genes with extreme Boolean scores. By selecting the 50% most extreme genes captures 90% of all cancer genes.

The second step of the validation consisted of a standard cross-validation schema by which a random 4/5 of the cancer genes comprised the training sample used to build the GBA to be tested against the remaining 1/5 of the cancer genes (testing sample). After repeating this process 1000 times, each with a different 4/5 training/1/5 testing random samples, we found that a ranked list of candidate genes comprising the top 13.2% of genes guarantees a 2.71-fold over-representation of cancer genes (Figure 4A). We also found that selecting the 50% most extreme genes, captures 90% of all cancer genes (Figure 4B).

When the subject is concerned with the identification of differentially expressed genes after normalising the data, one can invoke the Gaussian distribution to produce p-values. Similarly, when the issue is to ascertain enrichment of a particular biological process, one could invoke the hypergeometric distribution to produce p-values.

However and quite importantly, no parametric distribution functions were invoked in the development of the Boolean logic and the subsequent guilt-by-association algorithm. Instead, the sensitivity of the proposed approach in terms of its power to detect cancer genes was explored using a two-step procedures comprised of first assessing its efficiency when applied to cancer-associated genes, and then developing a cross-validation schema to identify the threshold beyond which the power to detect candidate genes is higher than the one obtained with known cancer-associated genes.

### The emergence of ranked candidate genes from the GBA algorithm

Table 2 lists the top 20 candidate genes and Additional File 4 contains the entire ranked list of 134 candidate genes (or 13.2% of the 1017 genes processed through the GBA). While a detailed description of the individual genes is beyond the scope of this study, we focus on candidates that also figure in the network analysis section described later, based on their connectivity to cancer-related genes and their position in the co-expression network.

**Table 2:** The top candidates identified by the GBA algorithm (genes with similar functional attributes are clustered together)

Candidate Genes	Normal	Adenoma	Carcinoma	Inflammation	Condition Specificity	Colon tissue specificity	Secreted Proteins	Transcription Factors	Protein kinases	PTMs	DNA Methylation
*GUCA2B*	11.01	5.66	7.52	8.05		✓	✓			✓	✓

*MMP1*	6.35	9.2	10.28	10.48			✓			✓	✓

*PAPPA*	6.51	5.88	7.71	7.12	✓	✓	✓			✓	✓

*PYY*	10.14	4.76	6.87	8.21	✓	✓	✓			✓	✓

*REG1A*	5.71	10.87	10.8	12.17	✓	✓	✓			✓	

*MEF2C*	8.66	7.36	8.43	9.04				✓			

*SOX2*	4.18	3.39	4.61	3.89		✓		✓			✓

*SPIB*	9.11	6.15	6.76	8.26	✓			✓		✓	✓

*WWTR1*	8.31	7.22	8.69	8.78				✓		✓	✓

*ZIC2*	2.22	4.8	3.53	2.55		✓		✓			✓

*CDK8*	8.62	8.75	8.96	8.29				✓	✓		

*EPHB3*	8.58	9.97	8.63	8.12	✓				✓	✓	✓

*ROR2*	5.16	4.4	5.47	5.56					✓		✓

*NPR1*	5.02	3.36	4.42	4.71	✓				✓	✓	

*TRIB3*	6.93	8.76	9.01	7.84					✓		

*TRPM6*	10.54	6.27	8.04	7.08	✓	✓			✓		

*GCG*	10.42	6.24	7.69	9.55	✓	✓	✓			✓	

*REG3A*	4.95	10.34	10.1	11.19	✓	✓	✓			✓	

*SERPING1*	8.9	8.11	9.28	10.21		✓				✓	

*SLC4A4*	11.76	8.76	9.57	9.81	✓	✓				✓	

#### Excretory-Secretory proteins as diagnostic or prognostic biomarkers

ES proteins are particularly relevant in colorectal cancer because most colorectal cancers develop slowly; beginning as small benign colorectal adenomas that progress over several years to larger dysplastic lesions that eventually become malignant. A total of 178 genes encoding ES proteins were found using this approach, of which 51 genes were tissue-specific to the colon. 64 entries had evidence for a PTM and 25 genes showed methylation in colon cell lines. Among these, we highlight *PYY *and *GUCA2B. PYY *(peptide YY) is a gut hormone highly expressed in the colon [20] and down regulated eight-fold in adenomas compared with the normal colon (Table 2). Its distinct variation in expression levels in the colon and gut region (gastric mucosa and rectum) compared with the cancerous colon makes it an important candidate gene for detailed biochemical characterization. As *PYY *is down regulated in carcinoma, it is unlikely candidate for early detection as decreased levels of protein in the cancer would not alter levels in the peripheral blood. *GUCA2B *(Uroguanylin) is a physiological regulator of intestinal fluid and electrolyte transport, 8-fold down regulated in adenoma, and its expression is observed in blood and urine [21]. Therefore, *GUCA2B *could be exploited as a non-invasive biomarker for the early detection of colorectal cancer.

#### Transcription factors as novel oncogenic regulators for the treatment for colorectal cancer

The altered activity of a few key TFs results in aberrant expression of their target genes, which can eventually lead to tumor development. The combination of the GBA and regulatory impact factor (RIF) analyses yielded 58 TF genes. Thirty-eight of these TFs showed colon-specific expression, 19 genes had DNA methylation and 6 proteins encoded by TFs had evidence for at least one PTM (Table 2). Here, we highlight the biological relevance of the top two candidates: *SPIB *and *MEF2C. SPIB *is an adenoma condition-specific down regulated gene. The DNA-binding ETS domain of *SPIB *is highly homologous to the ETS domain from the oncoprotein Spi-1/PU.1 [22] and may be an oncogenic TF awaiting experimental characterization. In addition, *SPIB *interacts with the promoter region of the c-JUN oncogene and *MAPK3 *gene [23] that are implicated in several cancers, including ovarian cancer. Similarly, *MEF2C *has been proven to play a role in angiogenesis [24], and shown to be over-expressed in hepatocellular carcinoma [25].

#### Genes encoding protein kinases

A total of 11 genes encoding protein kinases were identified of which 2 were tissue-specific and 3 genes were DNA methylated: *EPHB3, NPR1 *and *TRPM6. EPHB3 *is a receptor tyrosine kinase that mediates several developmental processes [26]. Importantly, *EPHB3 *interacts with the *Fyn *oncogene in vivo, and *EPHB3 *has a suggested role in tumor suppression. Other kinases predicted by the GBA include *NPR1*, a novel guanylate cyclase that catalyzes the production of cGMP from GTP and *TRPM6*, also called channel kinase 2, which is significantly down regulated in adenomas.

#### Post-Translational Modifications

PTMs such as glycosylation also go awry in cancer cells. This is seen as a result of the initial oncogenic transformation and a key event in the induction of invasion and metastasis in cancer [18]. By treating PTMs of proteins as a separate functional attribute in the Boolean logic, we found a total of 158 genes whose protein product harbors at least one PTM. A total of 32 entries with a PTM were tissue-specific with four overlapping the kinase set and 64 being secreted proteins, some of which had already been described in the previous sections. *REG3A*, a secreted protein that undergoes a proteolytic cleavage (a form of PTM) is up-regulated in adenomas, and could be a potential biomarker for the early detection of colorectal cancer.

#### DNA methylation as an epigenetic modification

DNA methylation (DNAm) patterns are altered in cancer cells, as shown by the hypomethylation of oncogenes and hypermethylation of tumor suppressor genes resulting in gene silencing and gene inactivation respectively [27,28]. Using genome-wide DNA methylome data for colon, we obtained 99 genes from the GBA algorithm as methylated genes. 17 of these genes have a preference for colon tissue expression and 19 of them were transcription factors, 23 proteins with a PTM and 22 secreted proteins. The *ADAMTS16, GUCA2B, PYY *and *THBS2 *genes were hypomethylated, whereas *FXYD1 *and *WWTR1 *were hypermethylated [29]. DNAm information can serve as additional evidence for these genes as potential candidate genes and should be further investigated.

### Gene co-expression networks reveal novel associations between cancer and candidate genes

It is thought that co-expressed genes are co-regulated by similar regulatory mechanisms; hence, possible functional collaborations between co-expressed genes have been proposed. To obtain a holistic view of the relationship between known and novel genes identified by the GBA algorithm, we constructed a series of gene co-expression networks using highly correlated differentially expressed and condition specific genes. Each network contained 1347 genes including the 530 cancer-associated genes and the 817 candidate genes that were captured by at least one of the seven expression-based variables (differentially expression or condition specificity). Of the 1 617 503 correlations evaluated in each network, the proportion found to be significant (referred to as clustering coefficient) according to PCIT algorithm and varied from 4.6% for the Adenoma network to 11.7% for the Carcinoma network (Table 3). The nodes (genes) and edges (connections) which were conserved in three or more network were retained to build what we referred to as the 'always-conserved network'.

**Table 3:** The properties of network connectivity:

	Normal	Adenoma	Carcinoma	Inflammation
Normal	5.18	2.28	3.31	4.25

Adenoma	1.20	4.63	8.26	5.25

Carcinoma	2.01	3.89	11.67	11.07

Inflammation	2.30	1.96	4.01	11.10

Clustering coefficients (%, on diagonals) and percent overlap computed from the ratio of common links divided by the total number of unique links for positive (above diagonal) and negative (below diagonal) links across each pair-wise network comparison.

**Figure 5:**
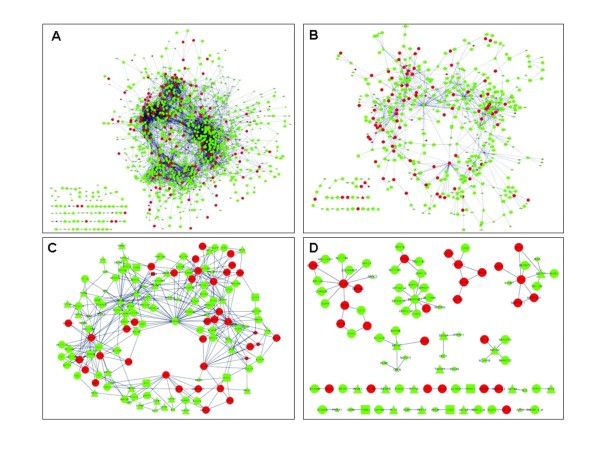
**The Always Conserved network visualized using the Cytoscape software at our levels of resolution**: (A) Connections involving at least one top candidate gene; (B) derived from A where only genes with more than two connections are displayed; (C) derived from B where only connections that were deemed to be significant across the four original networks (Adenoma, Carcinoma, Inflammation and Normal) are displayed; and (D) only those connections involving at least one top candidate gene in the four networks. The specific nature of edges, nodes and other features such as shape and color along with the Cytoscape file is provided in our website http://www.livestockgenomics.csiro.au/courses/crc.html

The always-conserved network shown in Figure 5 was further dissected into eight different networks and investigated for their properties. The first four networks were built in such a way that all the functional attributes were included. In essence, the first network (Figure 5A) represents pairs of genes connected in (i) all four networks, (ii) all four networks except Normal or (iii) all four networks except Carcinoma. The second network (Figure 5B) retains only those connections involving at least one top candidate gene. In the third network (Figure 5C), connections involving at least one top candidate gene where both genes have more than two connections are retained. Finally, the fourth network (Figure 5D) contains the least number of nodes among those connections involving at least one top candidate gene with a significant connection in all the four networks. The remaining four networks were constructed based on similar functional attributes. For instance, the TF-TF only (nodes: 49, edges: 37) network was built, in which only those connections where a transcription factor is connected to another transcription factor are retained. Similarly, other networks based on the post-translational modifications (nodes: 216, edges: 372), secreted proteins (nodes: 135, edges: 346) and kinases (nodes: 7, edges: 4) were built. The always-conserved networks are scale-free networks and the connectivity of the network follows a power-law distribution (Additional File 1 Figure S1). We addressed four key questions in the network analysis section: (i) which of the top candidate genes are hub genes? (ii) are there novel functional links between cancer and non-cancer-associated genes? (iii) are there any highly connected gene modules functionally relevant to cancer? and (iv) what is the nature of the attribute networks (TF-TF, SEC-SEC etc)?

Our network analysis identified a number of hub genes including several top candidate genes (Figure 5D). A notable, high impact module with *GUCA2B *as a hub gene with 41 connections is significant (Figure 5A). *GUCA2B *was connected to other top candidates such as *GUCA2A, CHGA *and importantly the nuclear receptor *NR3C2*, which is highly implicated in leukemia [30], colorectal carcinoma [31], and other carcinomas. Interestingly, *CHGA *was found to be the central link between two modules, one with *GUCA2B *as a hub and another module where *PYY, GCG *and *CHGB*, all candidate genes, were connected. Because these connections are based on significant correlations between gene pairs, they provide the first insights towards functional collaborations among the candidate genes found in this study. A number of network relationships were found among cancer-associated and non-cancer-associated genes. The MMP2 gene product which promotes tumor progression and metastasis by the degradation of the extra-cellular matrix [32] was connected to genes encoding candidate secreted proteins, *C1 S *and *COL5A1*.

We further explored functional relationships between cancer-associated and non-cancer associated genes by conducting enrichment analysis of GO categories using the BiNGO plug-in [33]. Among the top ten over-represented GO terms were anatomical structure development, immune response, response to stress and negative regulation of biological process. Notably, over-representation of GO category of importance from the colorectal cancer viewpoint is the inflammatory response, as chronic inflammation is widely believed to be a predisposing factor for colorectal cancer particularly in individuals with inflammatory bowel diseases; however the underlying molecular links between these two conditions have remained elusive. The only documented example is the role of STAT3 that links inflammation to tumor development in colorectal cancer [34]. Therefore, our list of candidate genes (*C1 S, CXCL11*, and *REG3A*) where inflammatory response is over-represented can be considered as potential candidates for elucidating unresolved cellular mechanisms mediating this relationship in colorectal cancer.

Next, we applied a combination of the BiNGO and MCODE plug-ins to study over-represented GO categories in the sub-networks [35]. Overall, we found 23 sub-networks of which the scores of five sub-networks were significant (Additional File 1). The first sub-network comprised of 44 highly connected nodes and 78 edges (4 cancer-associated genes and 40 non-cancer associated genes). This cluster was over-represented by GO terms, phosphate transport and response to external stimulus (that includes candidate genes *FPR2 *and *S100A8*). The cluster also contains several collagen sub-unit genes (*COL4A1, COL3A1*, *COL1A2*, and *COL5A2*). Again, over-representation of cell adhesion was evident in the second cluster with membership from five cancer-associated genes including *MMP2*. These cell adhesion molecules bind to components of the extracellular matrix and up-regulation and down-regulation of candidate genes identified in this study may play a role in cancer invasion and metastasis by altering the ability of cells to adhere to surrounding cells and the extracellular matrix [36].

Finally, network analysis of similar functional attributes such as the transcription factors only network and the secreted proteins only network captured additional regulatory hot spots and secreted protein modules that were not predicted with significant scores previously (Additional File 5). These four networks are of great relevance, since they are correlated by similar expression patterns, have interrelated functional attributes and are candidate non-cancer associated genes. For instance, in the TF-TF network (Additional File 5 Figure S1C), the hub genes (*NR5A2, MEF2C*) could be seen as regulatory hot spots that control gene expression via regulation of transcription.

### The RIF (Regulatory Impact Factor) analysis

We have recently introduced a novel metric called RIF or 'regulatory impact factors' to measure the regulatory capacity of transcription factors from gene expression data alone [37]. RIF uses two different measures, RIF1 and RIF2, to predict key regulators (TF) in driving the phenotypically relevant component of a given co-expression network. The highest impact regulators (extreme RIF |z-score| > 2) resulting from the RIF1 and RIF2 analysis are documented in Additional File 1 Table S3. A few notable regulators with extreme scores include *SAP18, CDK8, NR3C1, NFYC, CEBPB, PHF19 *and *TEAD4*. Of particular interest was the accurate prediction of CDK8 as the second-most significant regulator, recently identified as a colorectal cancer oncogene that regulates beta-catenin activity [38]. Second, *CEBPB *was established as a target gene for regulation in myeloid cells transformed by the *BCR/ABL *oncogene and also has a suggested role in promoting tumor invasiveness. Other potential regulators predicted by RIF such as *EPC1, SAP18 *and *ZNHIT3 *have no previous link with cancer and therefore provide an opportunity for further investigation.

## Conclusions

The method introduced here is highly flexible and can be implemented for any cancer type in a rather straightforward manner. Tissue specificity is one of the variables in the Boolean combinatorial logic that will require updating with every cancer type. For instance, one could study breast or pancreas-specific genes and their association with cancer by applying this method. Nuclear receptors are considered to be ideal drug candidates for treating breast cancer. We also believe that this approach could be applied to study other hereditary diseases such as Alzeimer's and Down's syndrome, provided sufficient molecular attributes are available for the respective diseases. Importantly, the candidate genes described here are classified based on individual attributes. Hence, those genes that share a number of attributes could be ranked as more promising candidates than their counterparts. For instance, *PYY *is a differentially expressed, condition-specific, tissue-specific to the colon, encoded product is a secreted protein that harbors a PTM and the gene is DNA hypomethylated in a colon cancer cell line. Therefore, *PYY *could be considered as a 'master candidate' awaiting further biochemical characterization. Finally, we argue that this is a holistic approach that faithfully mimics cancer characteristics, systematically predicts plausible cancer-associated candidate genes and has universal applicability to the study and advancement of cancer research.

## Methods

### Gene expression data: Identification of differentially expressed and condition-specific genes

We used the gene expression data from the colorectal cancer study of Galamb et al. (2008) profiling the gene expression from tissue samples classified as one of the following four conditions: normal (n = 8 samples), adenoma (15), carcinoma (15) and inflammation (15). Using the MAS5 detection call utility, probes yielding an absent signal in all 53 hybridizations were removed. As a result, we retained a total of 2 897 775 expression intensity signals across 34 844 probes that were annotated to 21 892 unique human genes were available for further analysis.

For the identification of differentially expressed genes we explored three contrasts: 1. Carcinoma vs. Normal; 2. Carcinoma vs. Adenoma; and 3. Carcinoma vs. Inflammation. For each contrast and following previously described approaches [39], a combination of ANOVA models and mixtures of distributions were employed to normalize expression signals and to identify differentially expressed genes, respectively. In brief, for each of the four datasets, data normalization was achieved by fitting a parsimonious mixed-effect ANOVA model containing the main fixed effect of the hybridization and the random effects of gene, gene × experimental condition interaction, and residual error. After building and solving the ANOVA model, the difference between the normalized expression of a gene in the two conditions of the given contrast was computed as the measure of (possible) differential expression. Finally, differentially expressed genes were identified using a two-component normal mixture model with an estimated experiment-wise false discovery rate (FDR) of < 1%.

For the identification of condition specific genes, a measure of the condition specificity of each gene was obtained from the ratio of its expression in the *j*-th condition (*j *= 1 to 4 for normal, adenoma, carcinoma and inflammation) over its expression summed across all four conditions as follows:

$$CS_{ij}=\frac{x_{ij}}{\sum_{j=1}^{4}x_{ij}}$$

Following the above expression, four measures of condition specificity were computed for each gene, and a gene was set to be condition-specific for the *j*-th condition if its expression in the *j*-th condition was (1) above the average expression of all genes in the *j*-th condition; (2) greater than its expression in any of the other three conditions; and (3) such that CS*_ij _*was greater than three standard deviations of all other CS*_ij_*'s.

### Cancer-associated genes

We compiled a list of cancer-associated genes by manual curation of literature and web-based resources. More than 1% of all human genes are implicated in cancer via mutations, and these genes collectively form the basis of cancer biology [15]. These genes form the basis of our "cancer-associated genes" dataset. First, we obtained 437 representative cancer-associated genes from the Cancer Gene Census at the Sanger Centre http://www.sanger.ac.uk/genetics/CGP/Census/. Next, we retrieved a second list of cancer related genes from the Atlas of Genetics and Cytogenetics in Oncology [40]. A third list was collated from the disease association data of HPRD database [41] and based on high confidence protein expression entries in multiple cancer tissues. In addition, we surveyed the lists of genes reported in the following research and review articles: [15]; [42]; [43]; and [44]. Finally, we collated these datasets to a master list of 749 cancer-associated genes Additional File 2.

### Functional attributes

We retrieved expression data from massively parallel signature sequencing (MPSS) covering 182 719 tag signatures across 32 tissues [45]. The complete list of TFs was retrieved from BiblioSphere [46] in the Genomatix web site http://genomatix.de. The post-translational modification (PTM) data were downloaded from the most recent version of the Human Protein Reference Database (HPRD - Release 9). A list of 1 764 high-confidence secreted proteins was obtained from the secreted protein database [47]. A catalogue of 518 protein kinase genes was downloaded from [48,49]. A list of alterations in DNA methylation specific for colorectal cancer using DNAm was obtained from the human colon cancer methylome [29]. Datasets for functional attributes are provided in Additional File 2.

### The Boolean Logic and the Guilt-by-Association Algorithm

As detailed in Mukherjee *et al. *[10], a k-ary Boolean function is a function f: {0,1}^k ^{0,1} which maps each of the 2^k ^possible states of its binary arguments X = (X_1 _⋯ X_k_) to a binary state Y. Such a function can also be represented as a truth table. In our case, we considered a total of k = 13 variables in the Boolean logic: Three measures of differentially expression, four measures of condition specificity, and the six functional attributes (TS, TF, PTM, KIN, SEC, and MET). These were binarized (prototypically 0 and 1) and used to compute what it's known as the probabilistic truth table, where the probabilities were obtained from the proportion of cancer-associated genes presenting a particular profile of 0's and 1's across the 13 variables. Therefore, the probabilistic Boolean truth table assigns a probability value to each existing combination of Boolean variables. In our case, this probability was derived from the proportion of cancer-associated genes exhibiting that combination. This trained model was then used as a GBA algorithm applied to non-cancer related genes in the human genome.

The GBA algorithm proceeded as follows:

• The particular combination across the 13 Boolean variables observed for a given non-cancer gene of interest was decomposed into its roots.

• The probability associated with each root was captured from the probabilistic Boolean truth table.

• These probabilities were added to rank the importance of the non-cancer gene of interest as a novel candidate. We illustrate this concept with an example.

Let's consider a gene, *MEF2C*, being differentially expressed for the second contrast, TF, PTM and MET. Across the 13 variables, this is equivalent to the Boolean profile"0100000011001" which can be decomposed in the following 14 roots each associated with a probability value corresponding to the probabilistic Boolean truth table (Table 4). Probability values on the third column add to 0.58868 and this value is the Boolean score used in the ranking of *MEF2C *as a novel cancer-related gene.

**Table 4:** The Boolean probabilistic truth table for *MEF2C *gene

No	Binarized Boolean profile	Probability values
1	0000000000001	0.05094

2	0000000001000	0.23019

3	0000000001001	0.02453

4	0000000010000	0.10755

5	0000000010001	0.03396

6	0000000011000	0.07925

7	0000000011001	0.03019

8	0100000000000	0.01509

9	0100000000001	0.00377

10	0100000001000	0.00377

11	0100000001001	0.00189

12	0100000010000	0.00377

13	0100000010001	0.00189

14	0100000011000	0.00189

### Computational Validation of the analytical approach

We designed a two-step approach to ascertain the inferential validity of the proposed GBA. In the first step, we processed all genes through the Boolean logic using the previously developed probabilistic truth table and recorded how extreme the cancer genes were ranked relative to the other genes. The ratio of the average Boolean score given to cancer genes over the average score given to the other genes was used as the threshold to be applied for the calibration in the second step of the validation.

The second step of the validation consisted of a standard cross-validation schema by which a random 4/5 of the cancer genes comprised the training sample used to build the GBA to be tested against the remaining 1/5 of the cancer genes (testing sample). We repeated this process 1000 times, each with a different 4/5 training/1/5 testing random samples. In each iteration, the number of cancer genes captured in the top *x*-percentile (for *x *= 1,2....,100) was recorded and used as the measure of sampling distribution upon which to infer the size of the ranked list of candidate genes that guarantees the threshold obtained in the step one of the validation is met.

### Reconstruction of Gene Co-Expression Networks

The PCIT algorithm [50] was used to reverse-engineer four gene networks, one for each condition: Normal, Adenoma, Carcinoma and Inflammation. The networks were constructed in such a way that a gene pair was allowed in the network only if it was conserved in at least three out of four conditions. Therefore, we refer to these networks as the 'Always conserved networks' A network for each of the four conditions, Normal, Adenoma, Carcinoma and Inflammation, was constructed and integrated (intersect) to create four levels of resolution. The first network (1255 nodes, 5122 edges) was built to include the pairwise connections of the genes that were connected in all four networks. It addition, we also produced pair-wise connections of all genes except the Normal and Carcinoma genes, which enabled us to investigate exclusive interactions in Normal and Carcinoma sets. The second network (534 nodes, 5122 edges) retained only those connections involving at least one top candidate gene. The third network consisted of those connections involving at least one top candidate gene and where both genes had more than two connections (146 nodes, 367 edges). Finally, the fourth network contained those connections involving at least one top candidate gene found to be significant in the four networks (99 nodes, 79 edges). The remaining four networks were specific to the functional attributes. They were the transcription factors only, the secreted proteins only and so on where all of the nodes belonged to one functional attribute. Functional enrichment using GO was carried out using BiNGO plug-in [33] in Cytoscape. In this study, hypergeometric test was used to assess the statistical significance (p < 0.05) and the Benjamini & Hochberg False Discovery Rate (FDR) correction.

### Identification of key transcription factors

Once the gene networks were obtained we applied the regulatory impact factor (RIF) algorithm of [37] to identity the key regulators, with emphasis in those not previously described as related to cancer. RIF assigns an extreme score to those transcription factors that are consistently most differentially co-expressed with the highly abundant and highly differentially expressed genes (case of RIF1 score), and to those transcription factors with the most altered ability to predict the abundance of differentially expressed genes (case of RIF2 score).

## Competing interests

The authors declare that they have no competing interests.

## Authors' contributions

AR conceived and supervised the project. SHN and AR carried out the analyses and drafted the manuscript. Both SHN and AR read and approved the final manuscript.

## Supplementary Material

Additional file 1 **Additional text, tables and figures that describe the rationale behind choosing the functional gene attributes, cancer pathway analysis and gene co-expression network analysis**. The file contains additional text on rationale behind choosing the functional gene attributes, text on cancer pathway analysis, figures and tables on network connectivity and network analysis using MCODE, BINGO plug-ins and RIF analysis. Click here for file

Additional file 2 **The list of cancer associated genes and publicly available datasets on functional attributes used in this study**. The list includes cancer associated genes, kinases, transcription factors, secreted proteins, proteins that undergo post-translational modifications and genes with CpG island methylation. Click here for file

Additional file 3 **Probabilistic Boolean truth table**. The truth table constructed from 749 cancer associated genes. Click here for file

Additional file 4 **The list of genes ranked by guilt-by-association algorithm**. The list comprises of 138 ranked list of candidate genes. Click here for file

Additional file 5 **Additional network analysis figures**. Network analysis of similar functional attributes (the TF only network, the SEC only network, TF only network and PTM only network). Click here for file
