# Supplementary material for: A Boolean-based systems biology approach to predict novel genes associated with cancer: Application to colorectal cancer
Source: BMC Syst Biol. 2011 Feb 26;5:35. doi: 10.1186/1752-0509-5-35 (PMC3051904; doi:10.1186/1752-0509-5-35)
Supplement: Additional file 1 — Additional text, tables and figures that describe the rationale behind choosing the functional gene attributes, cancer pathway analysis and gene co-expression network analysis. The file contains additional text on rationale behind choosing the functional gene attributes, text on cancer pathway analysis, figures and tables on network connectivity and network analysis using MCODE, BINGO plug-ins and RIF analysis. [file 1752-0509-5-35-S1.PDF]

Additional File 1
A Boolean-based systems biology approach to predict novel genes associated with cancer: Application to colorectal cancer

Shivashankar H Nagaraj and Antonio Reverter
[1]Computational and Systems Biology, CSIRO Livestock Industries, Queensland Bioscience Precinct, 306 Carmody Rd., St. Lucia, Brisbane, Queensland 4067, Australia.

Rationale behind choosing the functional gene attributes

Following extensive literature and data mining on cancer biology, five functional attributes were over-represented in cancer genes and therefore chosen for this study: genes encoding protein kinases, secreted proteins and transcription factors, tissue specificity of the genes, methylated genes and proteins that harbor post-translational modifications. Supplementary Table S2 provides the list of genes within each attribute. These features were selected based also on the fact that there is a strong functional interconnection among them (therefore we see the overlapping of genes across attributes); these molecules collectively carry out cellular processes as an ensemble rather than independent entities. These features are selected also based on the fact that there is strong interconnection between them and often (therefore we see overlapping of genes), these molecules collectively carry out cellular process as an ensemble rather than independent entities. Box 1 summarizes the general characteristics of the functional attributes with a few prototypic examples of representative. The significance of each attribute in cancer is also discussed.

Kinases: Cancer drugs targeting signaling pathways via kinase inhibitors
Protein kinases play central role in regulating most cellular functions via signal transduction pathways that includes cell proliferation, cell cycle, cell metabolism, survival/apoptosis, DNA damage repair, and cell motility. Undoubtedly, they have emerged as key regulators of all aspects of cancer, including proliferation, invasion, angiogenesis and metastasis. More than 30% of cancer related genes are kinases and the most common domain that is encoded by cancer genes is the protein kinase domain [1]. Some examples of kinases known to be activated in cancer cells are c-Src, c-Abl, RAS, mitogen activated protein (MAP) kinase, phosphotidylinositol-3-kinase (PI3K), AKT, and the epidermal growth factor receptor (EGFR) [2, 3]. A classic example of using kinase inhibitors for cancer treatment is the drug imatinib in the treatment of patients with chronic myelogenous leukemia (CML) where the kinase activity of BCR-ABL fusion protein is inactivated. Furthermore, more than 40 kinase inhibitors are in clinical trials, making kinases the second most popular drug target class, after G-protein-coupled receptors [2].

Excretory-Secretory proteins: as diagnostic or prognostic biomarkers
Malignant tumors secrete increased levels of ES proteins (cytokines, growth factors, and hormones) into the extracellular matrix including blood, serum and urine. These soluble ES proteins are extensively used as non-invasive diagnostic or prognostic markers, in cancer management or as points of therapeutic intervention. ES proteins are particularly relevant in CRC as most CRCs develop slowly, beginning as small benign colorectal adenomas that progress over several years to larger and dysplastic lesions that eventually become malignant. This gradual progression provides multiple

opportunities for early detection and prevention by simple colonoscopy and polypectomy [4]. In fact, the usefulness of individual ES proteins are well studied in colorectal cancer [4, 5], ovarian cancer [6] and prostate cancer [7].

Oncogenic Transcription factors

The overactivity of transcription factors at different stages of cancer is well documented and novel treatment strategies have been suggested for targeted inhibition of oncogenic TFs [8, 18, 19]. Even though limited numbers of TFs are involved in cancer, key regulatory networks are perturbed by TF results in aberrant expression of genes leading to tumor development. As TFs tightly control gene expression, selectively blocking transcription in tumor cells is an attractive therapeutic strategy [1, 8]. While MYB oncogene is the prototypic example for TF in cancer [20], four families of TFs have emerged as important players and are validated targets in drug discovery phase for cancer therapy; NF-kappaB [9], AP-1 [8], STAT [21] and ETS transcription factors [10].

Tissue specific expression of genes

There are more than 200 different types of cancer and many of them are localized to a specific tissue such as breast, lung, liver, colon etc. Roughly 30% of human genes are tissue specific genes (preferentially expressed in a given tissue). In other words, if a cancer preferentially affects a particular tissue, the likelihood of successfully identifying the responsible gene/s will be increased by restricting the search to genes that are more highly expressed there than in other tissues. Moreover, we [22] and others [23, 24] have shown that heritable disease genes are over-represented among tissue-specific genes. Cancer being a genetic disease, tissue-specific gene expression data was considered as a key functional gene attribute in this study.

DNA Methylation as epigenetic modification

Cancer is equally regarded as an epigenetic disease as much as it is accepted as a genetic disease. DNA methylation is an epigenetic modification exquisitely controlled during early development in the normal cell resulting in distinct methylation patterns. However, methylation patterns are altered in cancer cells as shown in hypomethylation of oncogenes and hypermethylation of tumor suppressor resulting in gene silencing or gene inactivation [12, 13]. Many cellular pathways are inactivated by this type of epigenetic lesion: apoptosis (DAPK), DNA repair (BRCA1, MGMT), cell cycle (p16(INK4a), p14(ARF)), cell adhesion (CDH1, CDH13), detoxification (GSTP1), [14] etc. There is mounting evidence to support that the alteration in DNA methylation is a hallmark of cancer and this feature could be used for diagnostic and therapeutic applications [15, 16]. Although, the list of genes aberrantly methylated in cancer is growing, recent genome scale analysis of human colon cancer methylome [11] provide an excellent coverage of methylation data for over 3000 human genes that is selected for this study.

Post-translational modification (PTM)

Post-translational modifications at the protein level are principal regulatory mechanism in eukaryotes and key proteins driving oncogenesis, can undergo PTMs. Experimentally, PTM data are not obtainable by gene arrays and therefore, this attribute complements genomic data from gene expression and links kinase singaling pathways into this analysis. Although phosphorylation is partially covered in kinases

section, other PTMs such as glycosylation [17] and ubiquitination [25] reported to play a role in malignancies, are included separate functional gene attributes.

Cancer Pathways
Genetic and epigenetic defects in cancer related genes automatically lead to dysregulation of respective signalling pathways. A number of key signaling pathways are also referred to 'cancer pathways' as defects in these pathways allow cancer cells to alter their normal programmes of proliferation, growth, migration, differentiation and death. Aberrant activation of cancer pathways has been linked to number of human malignancies including leukemias, lymphomas and carcinomas of the breast, colon, esophagus, pancreas, prostate, cervix and kidneys. Previous studies have focussed on specific pathways where only one of few candidate genes have been measured either for a potential biomarker or as a drug target [26]. However, in this study we have integrated comprehensive pathway data for all the available cancer signalling pathways that are generally applicable to cancer. Our data comprises of ten cancer signaling pathways (with 768 genes) from a publicly available resource, NetPath which is a manually curated resource of cancer signal transduction pathways. They include EGFR (Ras/Raf/MAPK signaling modules are included), NF-kB, Wnt/B-catenin, TGF-Beta receptor (TP53 and Rb pathways are included in TGFBR) and Notch signaling pathway. Additional information for each pathway and their role in various cancer types can be found at http://www.netpath.org

Results and Discussion

Targeting cancer pathways to discover novel genes in colorectal cancer: Defects in key cell-signaling pathways re-program biological processes, including those of proliferation, transcription, growth, migration, differentiation and death. The roles of several cancer-pathway genes remain incompletely explored in different cancer types, especially in CRC. Therefore, we took an independent approach to analyze cancer pathways (TGF beta Receptor, NOTCH, WNT pathways etc) and integrated them with the candidate genes from the current study. In order to predict potential candidate genes for colorectal cancer using the cancer pathway, we simply integrated the top differentially expressed or condition specific genes that were associated with well known cancer pathways. A total of 17 candidate genes were identified (Table 3). The highest number of candidate genes were present in the EGFR pathway (GJA1, KRT7, RGS16, SH2D3C, SOCS3), followed by four genes (HES5, HES6, MEF2C, NOTCH3) in the NOTCH signaling pathway. Finally, the GAS1 or Growth arrest-specific1 gene was predicted from the Hedgehog pathway. GAS1 is considered a putative tumor suppressor gene, and its role in metastasis is shown in melanoma cells. We observed a three-fold increase in GAS1 expression from adenoma to carcinoma, and therefore it is an important candidate that warrants further experimental characterization. The cancer pathways were treated differently in this analysis as members of cancer pathways themselves are important enough to host a number of potential candidates due to their documented role in cancer progression, and support from other functional attributes may be less relevant. These results should compliment other similar studies [27] where *in-silico* strategies have been used to map the patterns of oncogenic pathways from expression profiles from colorectal cancer patients.

Table S1: The top candidate genes involved in cancer pathways

**Candidate Genes**	**Normal**	**Adenoma**	**Carcinoma**	**Inflammation**	**CS1**	**CS2**	**CS3**	**CS4**	**Colon tissue specificity**	**TF**	**PTM**	**KIN**	**ES**	**MET**	**P-value**	**Cancer Pathway**
BTK	6.66	5.12	6.28	7.59	0	0	0	0	0	0	1	1	0	0	0.33	Kit Receptor
CLCA1	13.08	12.62	11.35	12.56	0	0	0	0	1	0	0	0	1	0	0.15	A6B4 Integrin
GAS1	2.86	2.68	6.80	6.22	0	0	0	0	0	0	0	0	0	0	0.03	Hedgehog
GJA1	9.45	9.18	10.22	10.20	0	0	0	0	0	0	1	0	0	0	0.25	EGFR
HES5	6.10	6.74	5.26	5.25	0	0	0	0	0	1	0	0	0	0	0.13	NOTCH
HES6	7.04	8.39	7.65	6.36	0	1	0	0	0	1	1	0	0	0	0.42	NOTCH
KRT7	2.78	5.76	4.48	6.38	0	0	0	0	0	0	1	0	0	0	0.27	EGFR
MEF2C	8.66	7.36	8.43	9.04	0	0	0	0	0	1	1	0	0	1	0.59	NOTCH
MEF2C	8.66	7.36	8.43	9.04	0	0	0	0	0	1	1	0	0	1	0.59	TGFBR
MMP7	6.32	9.35	8.66	9.54	0	0	0	0	0	0	1	0	1	0	0.31	A6B4 Integrin
NOTCH3	6.62	7.16	8.24	8.05	0	0	0	0	0	0	1	0	0	0	0.26	NOTCH
RGS16	5.14	6.35	6.64	6.52	0	0	0	0	0	0	1	0	0	0	0.24	EGFR
ROR2	5.16	4.40	5.47	5.56	0	0	0	0	0	0	0	1	0	1	0.09	WNT
SH2D3C	5.90	5.54	7.12	7.53	0	0	0	0	1	0	0	0	0	0	0.08	EGFR
SH3GL2	4.69	3.05	3.14	3.38	0	0	0	0	1	0	0	0	0	1	0.13	EGFR
SOCS3	6.15	7.65	8.43	9.11	0	0	0	0	0	0	1	0	0	0	0.24	EGFR
SOX1	3.39	5.30	3.90	2.99	0	0	0	0	0	1	0	0	0	1	0.22	WNT
STAP1	6.08	4.48	5.36	7.06	0	0	0	0	0	0	0	0	0	0	0.00	Kit Receptor

Methods:

The Identification of Differentially Expressed Genes
Let x_{ij} indicate the MAS5 expression intensity signal of the i-th gene averaged across all samples from the j-th condition where j = 1, 2, 3, and 4, for Normal, Adenoma, Carcinoma, and Inflammation, respectively. Contrasting x_{i3} for Carcinoma against each of the other three conditions, we considered three measures of differential expression (DE) as follows:

1. Carcinoma *versus* Normal: $DE1_i = x_{i3} - x_{i1}$
2. Carcinoma *versus* Adenoma: $DE2_i = x_{i3} - x_{i2}$
3. Carcinoma *versus* Inflammation: $DE3_i = x_{i3} - x_{i4}$

Following [27], a two-component normal mixture model was fitted to identify the DE genes.

$$f(\mathbf{d};\Phi) = \pi_0\,\phi_0\left(\mathbf{d};\mu_0,\sigma_0^2\right) + \pi_1\,\phi_1\left(\mathbf{d};\mu_1,\sigma_1^2\right),$$

where $\mathbf{d}$ denotes the vector of DE measures for all of the genes, and the two components in the mixtures correspond to: $\phi_0(\bullet)$ for the empirical null normal density with the mean μ_0 (not necessarily zero) and variance σ_0^2 (not necessarily one), encapsulating the non-DE genes; and $\phi_1(\bullet)$ for the non-null distribution corresponding to the DE genes. Finally, the mixing proportions π_0 and π_1 were constrained to be non-negative and sum to unity. Across the four datasets, the parameters of the mixture model were estimated using the EMMIX-GENE software [28].
Using an estimated experiment-wise false discovery rate (FDR) of < 1%, we found 444, 658 and 179 DE genes for DE1, DE2, and DE3, respectively.

Gene expression data:
The original raw data (CEL files) for the 53 chips were normalized and log2-transformed using the MAS5 algorithm (Affymetrix) available in the Bioconductor package simpleaffy. Using the MAS5 detection call utility, the probes yielding a marginal or absent signal in all 53 hybridizations were removed from further analyses. As a result, we retained 1,846,732 expression intensity signals across 34,844 probes that were annotated to 21,892 unique genes.

Cancer pathways: Cancer pathways
Ten cancer-signaling pathways with their respective gene members were downloaded from the NetPath database [48]. NetPath is a manually curated resource of signal transduction pathways in humans. Data for the following cancer pathways were downloaded: Alpha6 Beta4 Integrin (55), Androgen Receptor (92), EGFR (177), Hedgehog (21), Inhibitor of DNA binding (ID) pathway (28), Kit Receptor (65), NOTCH (79), TGFBR(153), TNF-alpha/NF-kB (189) and WNT Signaling Pathway (105). A total of 768 unique genes were included in this study. A total of 768 unique genes were included in this study.

The Always Conserved Network Analysis
Power law distribution of network connectivity in Always Conserved network

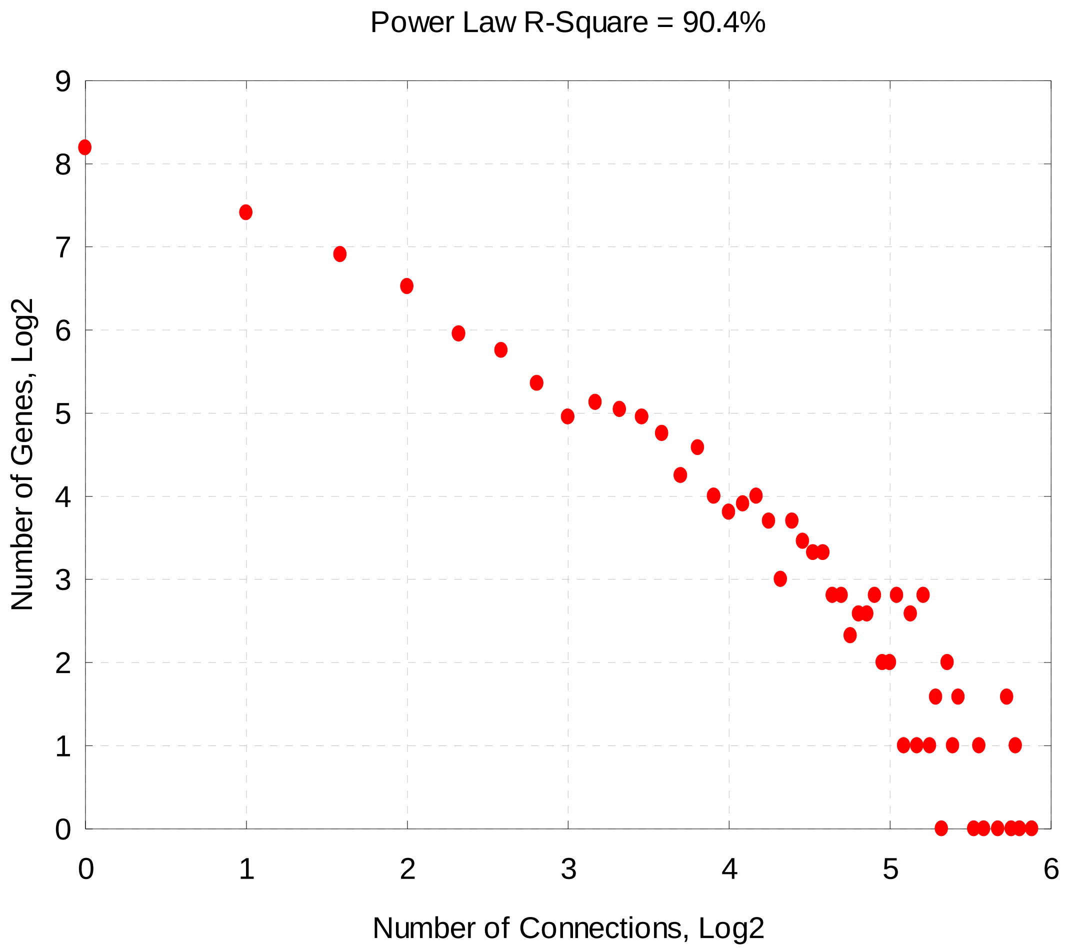

Top 30 hub genes in the always conserved network

Gene ID (Nodes)	Number of Connections (Edges)	Gene ID (Nodes)	Number of Connections (Edges)
CDKN2B	56	CD93	41
GPR116	55	CDH5	41
SERPINE1	55	GUCA2B	41
CD248	54	MGC13057	41
NR5A2	53	DHRS11	40
SLC4A4	53	CALD1	39
SPARC	53	CCDC3	39
BGN	51	COL1A2	39
COL4A1	48	HHLA2	38
ITGA5	47	LOXL2	38
PKIB	47	ARL14	37
COL15A1	46	CTHRC1	37
COL5A2	43	EDNRA	37
FCGR3B	43	IL8RB	37
HPGD	43		
COL6A3	42		

Analysis of Always conserved gene co-expression networks: sub-networks predicted from MCODE and GO enrichment from BINGO plug-ins. Over-represented GO categories for each sub-network with statistically significant p-values are shown.

Sub-network 1:

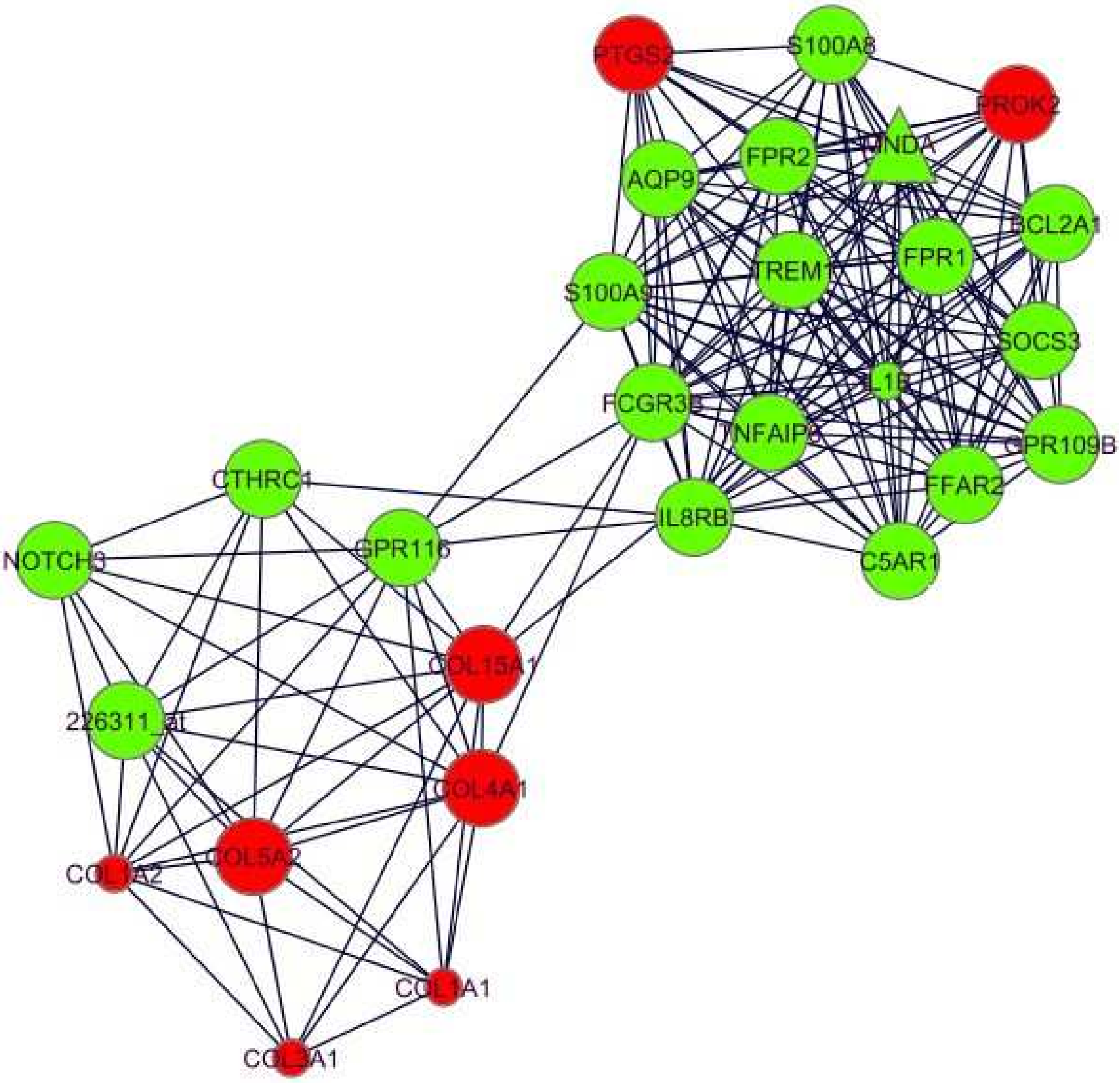

Over-represented GO categories

GO-ID	p-value	Description	Genes in the sub-network
6817	2.40E-10	phosphate transport	CTHRC1,COL4A1,COL3A1,COL1A2,COL15A1,COL1A1,COL5A2
9605	5.03E-09	response to external stimulus	TNFAIP6,IL8RB,PROK2,C5AR1,S100A8,S100A9,COL3A1,FPR1,IL1B,COL1A1,FPR2
42221	5.12E-09	response to chemical stimulus	IL8RB,PROK2,C5AR1,PTGS2,AQP9,S100A9,COL3A1,FPR1,IL1B,COL1A1,FPR2
6935	5.54E-09	chemotaxis	IL8RB, PROK2, C5AR1, S100A9, FPR1, IL1B, FPR2
15698	1.27E-08	inorganic anion transport	CTHRC1,COL4A1,COL3A1,COL1A2,COL15A1,COL1A1,COL5A2

Sub-network 2:

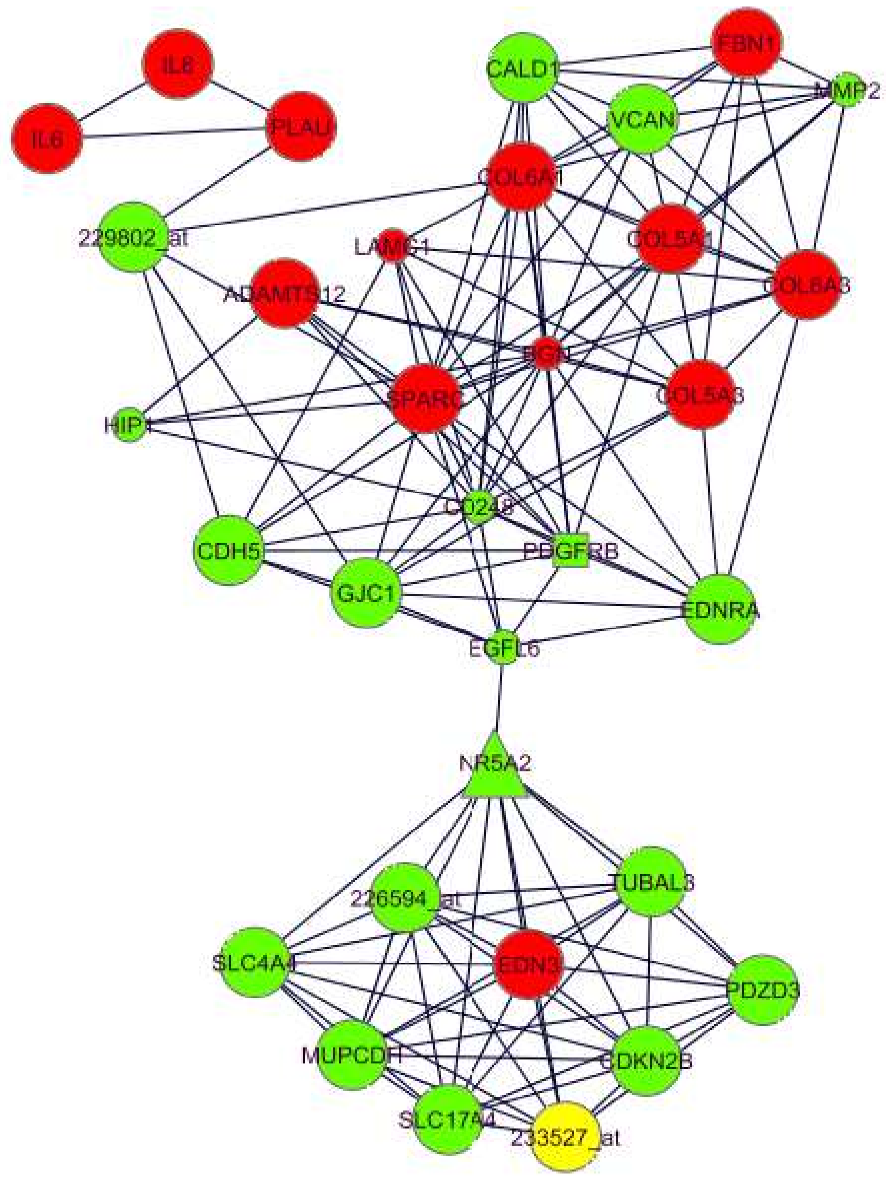

Over-represented GO categories

GO-ID	p-value	Description	Genes in the sub-network
7155	5.09E-06	cell adhesion	MUPCDH,EGFL6,COL6A3,COL6A1,VCAN,LAMC1,COL5A3,COL5A1,CDH5
6820	4.17E-05	anion transport	COL6A3,COL6A1,COL5A3,SLC4A4,COL5A1
51179	2.07E-04	localization	EDN3,IL8,CALD1,COL5A3,COL5A1,GJC1,EDNRA,SLC17A4,COL6A3,TUBAL3,COL6A1,LAMC1,SLC4A4,PDZD3,HIP1
32502	4.55E-04	developmental process	EDN3,IL6,IL8,EGFL6,FBN1,SPARC,COL5A3,MMP2,GJC1,COL6A3,VCAN,LAMC1,NR5A2,HIP1
7275	5.64E-04	multicellular organismal development	EDN3,IL8,EGFL6,FBN1,COL6A3,VCAN,LAMC1,SPARC,NR5A2,COL5A3,MMP2,GJC1

Sub-network 3:

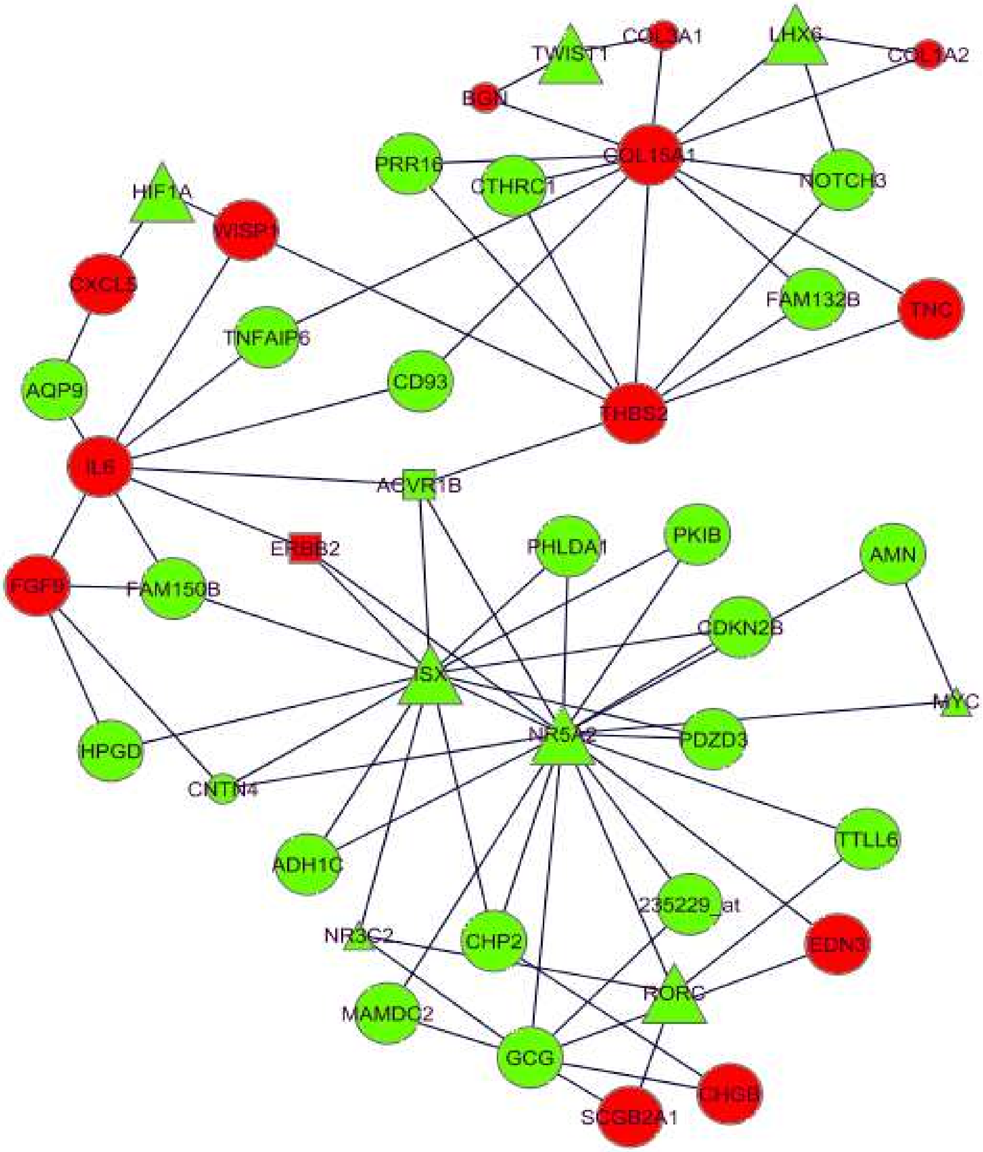

Over-represented GO categories

GO-ID	p-value	Description	Genes in the sub-network
48519	2.65E-06	negative regulation of biological process	IL6,FGF9,ERBB2,COL3A1,NOTCH3,ACVR1B, HIF1A,CDKN2B,CNTN4,MYC,PDZD3,HPGD,TWIST1
48523	8.86E-06	negative regulation of cellular process	NOTCH3,ACVR1B,IL6,HIF1A,CDKN2B,FGF9,ERBB2,CNTN4,HPGD,PDZD3,MYC,TWIST1
8284	2.57E-05	positive regulation of cell proliferation	IL6, HIF1A, CXCL5, FGF9, ERBB2, MYC
7167	3.89E-05	enzyme linked receptor protein signaling pathway	ACVR1B,ERBB2,COL3A1,COL1A2,HPGD,PDZD3
9967	7.86E-05	positive regulation of signal transduction	ACVR1B,IL6,HIF1A,CDKN2B,FGF9
42221	1.30E-04	response to chemical stimulus	GCG,IL6,HIF1A,CDKN2B,AQP9,CXCL5,COL3A1,PDZD3
51242	1.65E-04	positive regulation of cellular process	NOTCH3,ACVR1B,EDN3,IL6,HIF1A,CDKN2B, CXCL5,FGF9,ERBB2,MYC
42127	1.84E-04	regulation of cell proliferation	IL6,HIF1A,CDKN2B,CXCL5,FGF9,ERBB2,MYC

Sub-network 4:

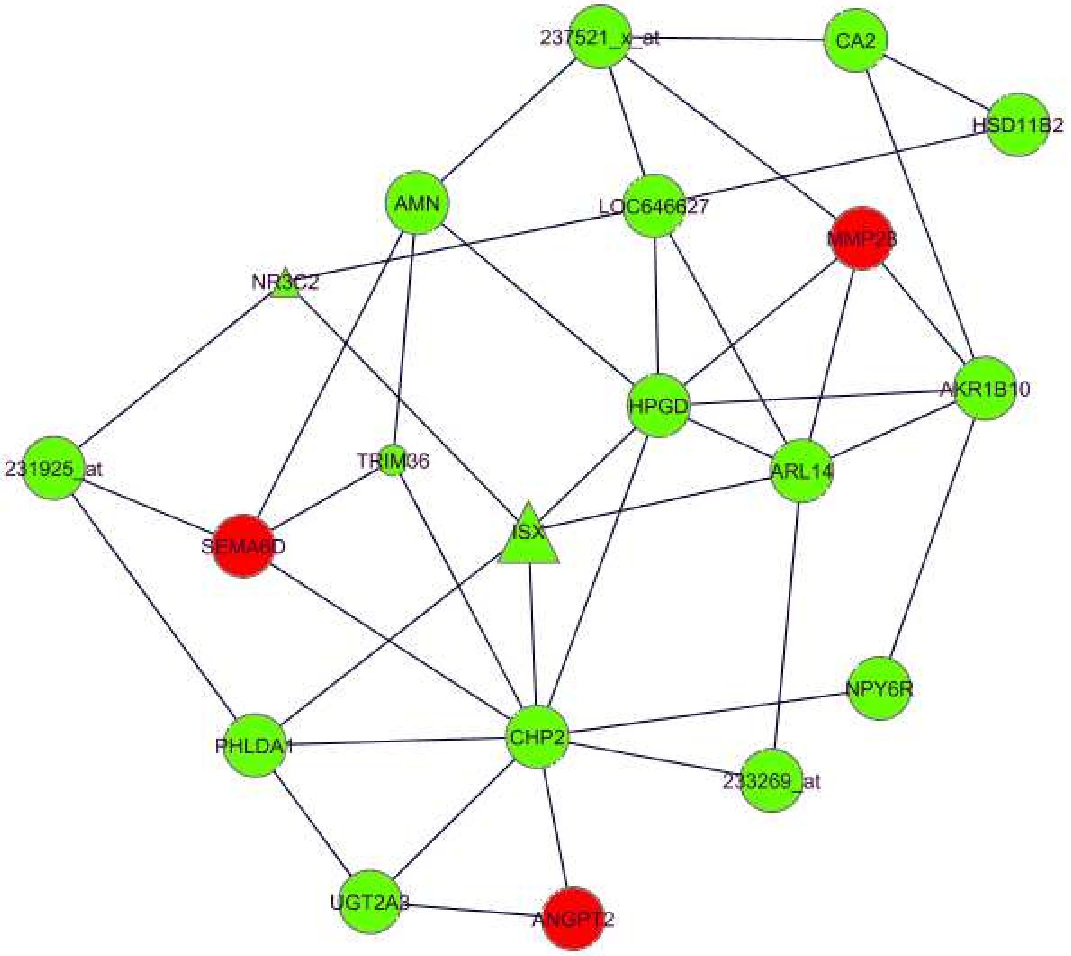

Over-represented GO categories

GO-ID	p-value	Description	Genes in the sub-network
44255	1.79E-03	cellular lipid metabolic process	SEMA6D,AKR1B10,HSD11B2,HPGD
8610	2.03E-03	lipid biosynthetic process	SEMA6D,HSD11B2,HPGD

Sub-network 5:

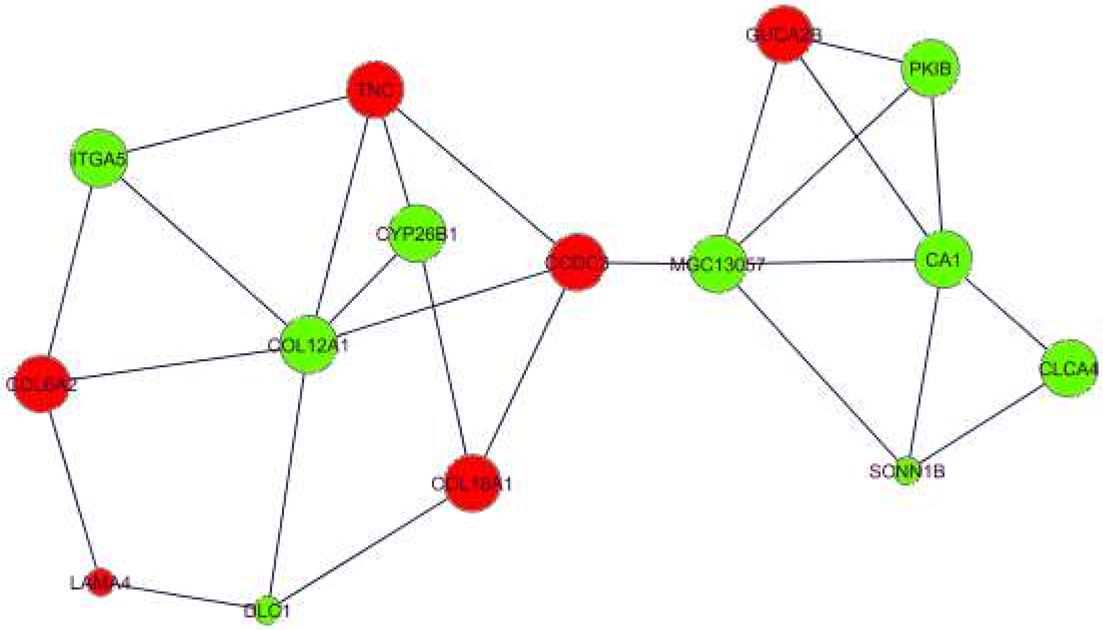

Over-represented GO categories

GO-ID	p-value	Description	Genes in the sub-network
7155	1.18E-05	cell adhesion	COL18A1,DLC1,ITGA5,TNC,COL6A2,COL12A1
43062	3.94E-05	extracellular structure organization and biogenesis	TNC,COL6A2,COL12A1
6817	6.50E-05	phosphate transport	COL18A1,COL6A2,COL12A1
15698	3.46E-04	inorganic anion transport	COL18A1,COL6A2,COL12A1
7588	5.94E-04	excretion	GUCA2B,SCNN1B
32501	2.82E-03	multicellular organismal process	COL18A1,DLC1,ITGA5,TNC,CYP26B1,COL12A1,GUCA2B,SCNN1B

Top Transcription Factors predicted by RIF 1 and RIF 2 algorithm

RIF1								
**Gene**	**RIF1**	**RIF2**	**TS**	**Functional Attributes**	**Normal**	**Adenoma**	**Carcinoma**	**Inflammation**
SAP18	-3.38	0.51	0	10000	11.24	11.46	11.37	11.07
CDK8	-3.22	1.56	0	10100	8.42	8.75	8.96	8.29
NR3C1	-3.16	0.31	0	11001	9.13	7.86	8.82	9.32
ZNHIT3	-3.11	1.38	0	10000	8.93	9.32	9.02	8.68
NFYC	-2.91	0.96	1	10000	8.93	8.71	8.62	8.70
KLF13	-2.80	1.99	0	11000	10.48	10.03	10.19	10.37
GATA3	-2.79	0.07	0	10001	7.49	6.78	7.21	7.86
ZNF43	-2.79	0.98	0	10000	8.37	6.57	7.51	7.68
RORC	-2.78	0.75	0	10000	7.70	8.00	6.91	7.13
PROX1	-2.72	-0.03	0	10001	7.70	8.85	7.76	7.47
TSC22D4	-2.70	0.38	0	10000	6.63	6.20	6.16	6.48
EPC1	-2.68	1.85	0	10000	9.55	9.22	9.21	9.49
BRPF3	-2.66	1.87	0	10000	8.36	7.56	7.97	7.81
RFX5	2.62	0.24	0	11000	8.02	8.50	8.40	8.70
SNAPC1	2.68	2.09	0	10000	6.33	7.14	6.93	7.02
HIF1A	2.70	0.73	0	11000	11.03	11.36	11.59	11.99
ESRRA	2.70	0.35	0	11000	9.95	9.64	9.44	9.31
KLF2	2.76	-0.10	1	10000	8.18	8.14	9.07	9.67
ELK3	2.91	1.28	0	11000	8.30	8.78	9.23	9.39
PHF13	3.06	1.85	0	10000	7.99	8.59	8.49	8.49
**RIF2**								
**Gene**	**RIF1**	**RIF2**	**TS**	**Functional Attributes**	**Normal**	**Adenoma**	**Carcinoma**	**Inflammation**
PIR	0.56	2.19	0	10000	6.79	7.10	7.13	7.33
CIP29	-0.03	2.19	0	10000	9.13	9.56	9.48	9.56
NR4A1	-0.24	2.21	0	11000	6.67	7.21	7.34	7.10
SAP30	1.13	2.21	0	11010	7.68	8.40	8.54	8.32
ZNF397	0.58	2.27	0	10000	8.02	7.82	7.50	7.52
MAFF	1.66	2.30	0	10000	8.00	9.09	9.05	9.57
PHF19	-0.42	2.31	0	10000	6.65	8.19	7.72	7.46
STRAP	-0.45	2.36	1	10000	10.89	11.38	11.33	11.07
KLF3	0.02	2.38	0	10000	11.16	10.76	10.93	10.61
MAML3	-0.14	2.40	0	10000	8.61	8.32	7.90	7.67
SETD7	0.91	2.42	0	10000	9.43	10.06	10.19	9.98
NR5A2	0.31	2.48	1	10001	9.46	7.65	8.34	8.56
DDX21	0.24	2.50	0	11000	10.13	11.22	10.93	10.45
SMAD7	-0.80	2.51	0	11000	9.46	8.93	8.67	8.67
GTF3A	-0.77	2.57	0	10000	10.48	11.11	11.07	10.80
TEAD4	-1.49	2.61	0	10000	6.22	7.65	7.43	7.17
TCEB1	-1.45	2.68	0	10000	10.08	10.55	10.62	10.21
NFE2L3	-1.68	2.73	0	10010	7.21	9.06	8.58	8.01
CEBPB	1.63	3.11	0	11000	10.34	11.27	11.63	11.62
NCOA7	0.28	3.19	1	10000	10.10	11.50	11.19	11.57

References:

1. Futreal PA, Coin L, Marshall M, Down T, Hubbard T, Wooster R, Rahman N, Stratton MR: **A census of human cancer genes**. *Nat Rev Cancer* 2004, **4**(3):177-183.
2. Dancey J, Sausville EA: **Issues and progress with protein kinase inhibitors for cancer treatment**. *Nat Rev Drug Discov* 2003, **2**(4):296-313.
3. Blume-Jensen P, Hunter T: **Oncogenic kinase signalling**. *Nature* 2001, **411**(6835):355-365.
4. Welsh JB, Sapinoso LM, Kern SG, Brown DA, Liu T, Bauskin AR, Ward RL, Hawkins NJ, Quinn DI, Russell PJ *et al*: **Large-scale delineation of secreted protein biomarkers overexpressed in cancer tissue and serum**. *Proc Natl Acad Sci U S A* 2003, **100**(6):3410-3415.
5. Buckhaults P, Rago C, St Croix B, Romans KE, Saha S, Zhang L, Vogelstein B, Kinzler KW: **Secreted and cell surface genes expressed in benign and malignant colorectal tumors**. *Cancer Res* 2001, **61**(19):6996-7001.
6. Welsh JB, Zarrinkar PP, Sapinoso LM, Kern SG, Behling CA, Monk BJ, Lockhart DJ, Burger RA, Hampton GM: **Analysis of gene expression profiles in normal and neoplastic ovarian tissue samples identifies candidate molecular markers of epithelial ovarian cancer**. *Proc Natl Acad Sci U S A* 2001, **98**(3):1176-1181.
7. Welsh JB, Sapinoso LM, Su AI, Kern SG, Wang-Rodriguez J, Moskaluk CA, Frierson HF, Jr., Hampton GM: **Analysis of gene expression identifies candidate markers and pharmacological targets in prostate cancer**. *Cancer Res* 2001, **61**(16):5974-5978.
8. Libermann TA, Zerbini LF: **Targeting transcription factors for cancer gene therapy**. *Curr Gene Ther* 2006, **6**(1):17-33.
9. Kim HJ, Hawke N, Baldwin AS: **NF-kappaB and IKK as therapeutic targets in cancer**. *Cell Death Differ* 2006, **13**(5):738-747.
10. Oikawa T: **ETS transcription factors: possible targets for cancer therapy**. *Cancer Sci* 2004, **95**(8):626-633.
11. Irizarry RA, Ladd-Acosta C, Wen B, Wu Z, Montano C, Onyango P, Cui H, Gabo K, Rongione M, Webster M *et al*: **The human colon cancer methylome shows similar hypo- and hypermethylation at conserved tissue-specific CpG island shores**. *Nat Genet* 2009, **41**(2):178-186.
12. Esteller M: **CpG island hypermethylation and tumor suppressor genes: a booming present, a brighter future**. *Oncogene* 2002, **21**(35):5427-5440.
13. Ehrlich M: **DNA methylation in cancer: too much, but also too little**. *Oncogene* 2002, **21**(35):5400-5413.
14. Esteller M, Herman JG: **Cancer as an epigenetic disease: DNA methylation and chromatin alterations in human tumours**. *J Pathol* 2002, **196**(1):1-7.
15. Szyf M: **Targeting DNA methylation in cancer**. *Ageing Res Rev* 2003, **2**(3):299-328.
16. Hoffmann MJ, Schulz WA: **Causes and consequences of DNA hypomethylation in human cancer**. *Biochem Cell Biol* 2005, **83**(3):296-321.
17. Hakomori S: **Glycosylation defining cancer malignancy: new wine in an old bottle**. *Proc Natl Acad Sci U S A* 2002, **99**(16):10231-10233.
18. Darnell JE, Jr.: **Transcription factors as targets for cancer therapy**. *Nat Rev Cancer* 2002, **2**(10):740-749.

19. Pandolfi PP: **Transcription therapy for cancer**. *Oncogene* 2001, **20**(24):3116-3127.
20. Introna M, Golay J: **How can oncogenic transcription factors cause cancer: a critical review of the myb story**. *Leukemia* 1999, **13**(9):1301-1306.
21. Haura EB, Turkson J, Jove R: **Mechanisms of disease: Insights into the emerging role of signal transducers and activators of transcription in cancer**. *Nat Clin Pract Oncol* 2005, **2**(6):315-324.
22. Reverter A, Ingham A, Dalrymple BP: **Mining tissue specificity, gene connectivity and disease association to reveal a set of genes that modify the action of disease causing genes**. *BioData Min* 2008, **1**(1):8.
23. Winter EE, Goodstadt L, Ponting CP: **Elevated rates of protein secretion, evolution, and disease among tissue-specific genes**. *Genome Res* 2004, **14**(1):54-61.
24. Lage K, Hansen NT, Karlberg EO, Eklund AC, Roque FS, Donahoe PK, Szallasi Z, Jensen TS, Brunak S: **A large-scale analysis of tissue-specific pathology and gene expression of human disease genes and complexes**. *Proc Natl Acad Sci U S A* 2008, **105**(52):20870-20875.
25. Burger AM, Seth AK: **The ubiquitin-mediated protein degradation pathway in cancer: therapeutic implications**. *Eur J Cancer* 2004, **40**(15):2217-2229.
26. Sasaki N, Morisaki T, Hashizume K, Yao T, Tsuneyoshi M, Noshiro H, Nakamura K, Yamanaka T, Uchiyama A, Tanaka M *et al*: **Nuclear factor-kappaB p65 (RelA) transcription factor is constitutively activated in human gastric carcinoma tissue**. *Clin Cancer Res* 2001, **7**(12):4136-4142.
27. McLachlan GJ, Bean RW, Jones LB: **A simple implementation of a normal mixture approach to differential gene expression in multiclass microarrays**. *Bioinformatics* 2006, **22**(13):1608-1615.
28. McLachlan GJ, Bean RW, Peel D: **A mixture model-based approach to the clustering of microarray expression data**. *Bioinformatics* 2002, **18**(3):413-422.
