# Supplementary material for: A Boolean-based systems biology approach to predict novel genes associated with cancer: Application to colorectal cancer
Source: BMC Syst Biol. 2011 Feb 26;5:35. doi: 10.1186/1752-0509-5-35 (PMC3051904; doi:10.1186/1752-0509-5-35)
Supplement: Additional file 3 — Probabilistic Boolean truth table. The truth table constructed from 749 cancer associated genes. [file 1752-0509-5-35-S3.PDF]

A Boolean-based systems biology approach to predict novel genes associated with cancer: Application to colorectal cancer

Shivashankar H Nagaraj and Antonio Reverter

Additional File 3: Probabilistic Boolean truth table constructed from 749 cancer associated genes

Binarized Boolean Combinations	Number of Cancer associated genes	P- value
0000000000001	27	0.0509434
0000000000010	19	0.0358491
0000000000011	3	0.00566038
0000000000100	9	0.0169811
0000000000101	4	0.00754717
0000000001000	122	0.230189
0000000001001	13	0.0245283
0000000001010	14	0.0264151
0000000001011	3	0.00566038
0000000001100	33	0.0622642
0000000001101	3	0.00566038
0000000001110	3	0.00566038
0000000001111	2	0.00377358
0000000010000	57	0.107547
0000000010001	18	0.0339623
0000000010100	2	0.00377358
0000000011000	42	0.0792453
0000000011001	16	0.0301887
0000000011100	1	0.00188679
0000000100000	32	0.0603774
0000000100001	5	0.00943396
0000000100010	1	0.00188679
0000000100100	1	0.00188679
0000000101000	16	0.0301887
0000000101001	4	0.00754717
0000000101010	2	0.00377358
0000000101100	3	0.00566038
0000000101111	1	0.00188679
0000000110000	2	0.00377358
0000000110001	5	0.00943396
0000000111000	4	0.00754717
0000000111001	1	0.00188679
0001000000001	1	0.00188679
0010000000000	1	0.00188679
0010000011000	1	0.00188679
0010000011001	1	0.00188679
0010000100000	2	0.00377358
0100000000000	8	0.0150943
0100000000001	2	0.00377358
0100000000010	4	0.00754717
0100000001000	2	0.00377358
0100000001001	1	0.00188679
0100000001010	3	0.00566038

0100000001110	1	0.00188679
0100000010000	2	0.00377358
0100000010001	1	0.00188679
0100000011000	1	0.00188679
0100000100000	4	0.00754717
0100000100001	1	0.00188679
0100000100010	1	0.00188679
0100000101000	1	0.00188679
0100000101010	1	0.00188679
0100100000000	1	0.00188679
0100100000001	1	0.00188679
0110000001011	1	0.00188679
1000000000000	5	0.00943396
1000000000001	1	0.00188679
1000000000010	1	0.00188679
1000000001000	2	0.00377358
1000000001110	1	0.00188679
1000000100001	1	0.00188679
1000100000001	1	0.00188679
1000100010000	1	0.00188679
1001000000000	1	0.00188679
1001000000010	1	0.00188679
1100000000000	1	0.00188679
1100000000010	1	0.00188679
1100000001010	1	0.00188679
1100000101011	1	0.00188679
1100010000010	1	0.00188679
