# Supplementary material for: A Boolean-based systems biology approach to predict novel genes associated with cancer: Application to colorectal cancer
Source: BMC Syst Biol. 2011 Feb 26;5:35. doi: 10.1186/1752-0509-5-35 (PMC3051904; doi:10.1186/1752-0509-5-35)
Supplement: Additional file 5 — Additional network analysis figures. Network analysis of similar functional attributes (the TF only network, the SEC only network, TF only network and PTM only network). [file 1752-0509-5-35-S5.PPT]

## Slide 1
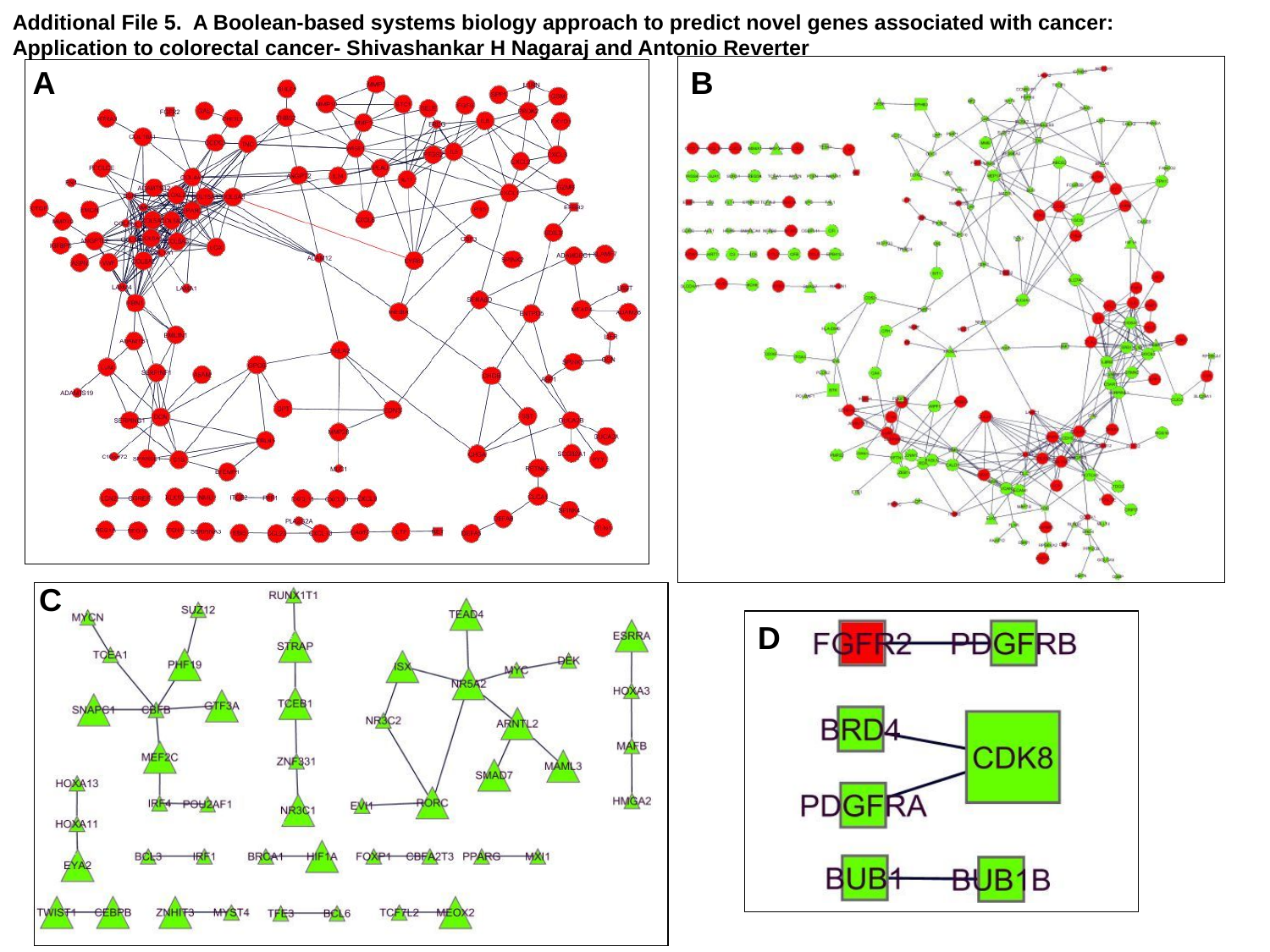

Additional File 5. A Boolean-based systems biology approach to predict novel genes associated with cancer: Application to colorectal cancer- Shivashankar H Nagaraj and Antonio Reverter
A
B
C
D
